# Virtual patients - what are we talking about? A framework to classify the meanings of the term in healthcare education

**DOI:** 10.1186/s12909-015-0296-3

**Published:** 2015-02-01

**Authors:** Andrzej A Kononowicz, Nabil Zary, Samuel Edelbring, Janet Corral, Inga Hege

**Affiliations:** 1 Department of Learning, Informatics Management and Ethics, Karolinska Institutet, Stockholm, Sweden; 2 Department of Bioinformatics and Telemedicine, Jagiellonian University Medical College, Krakow, Poland; 3 School of Medicine at the University of Colorado Denver in Denver, Colorado, USA; 4 Institut für Didaktik und Ausbildungsforschung in der Medizin am Klinikum der Universität München, Ziemssenstr 1, 80336 München, Germany

**Keywords:** Virtual patients, Healthcare education, Classification

## Abstract

**Background:**

The term “virtual patients” (VPs) has been used for many years in academic publications, but its meaning varies, leading to confusion. Our aim was to investigate and categorize the use of the term “virtual patient” and then classify its use in healthcare education.

**Methods:**

A literature review was conducted to determine all articles using the term “virtual patient” in the title or abstract. These articles were categorized into: Education, Clinical Procedures, Clinical Research and E-Health. All educational articles were further classified based on a framework published by Talbot et al. which was further developed using a deductive content analysis approach.

**Results:**

536 articles published between 1991 and December 2013 were included in the study. From these, 330 were categorized as educational. Classifying these showed that 37% articles used VPs in the form of Interactive Patient Scenarios. VPs in form of High Fidelity Software Simulations (19%) and Virtual Standardized Patients (16%) were also frequent. Less frequent were other forms, such as VP Games.

Analyzing the literature across time shows an overall trend towards the use of Interactive Patient Scenarios as the predominant form of VPs in healthcare education.

**Conclusions:**

The main form of educational VPs in the literature are Interactive Patient Scenarios despite rapid technical advances that would support more complex applications. The adapted classification provides a valuable model for VP developers and researchers in healthcare education to more clearly communicate the type of VP they are addressing avoiding misunderstandings.

**Electronic supplementary material:**

The online version of this article (doi:10.1186/s12909-015-0296-3) contains supplementary material, which is available to authorized users.

## Background

Virtual patients (VPs) have been used for many years in various contexts [1]: in healthcare education [2-4], electronic patient records [5-7], and clinical research [8-10]. Yet among these applications there exists a heterogeneous understanding about what virtual patients are and for what purposes they may be used. Particularly in the educational field the term “virtual patient” is applied to many diverse approaches [11-13].

Similar heterogeneity exists among definitions that are used to specify the term “virtual patient”. One recently proposed definition is quite general: “In the context of medical education, this term [VP] generally refers to any software that allows case-based training” [14].

An often-cited and more specific definition by the American Association of Medical Colleges (AAMC) delineates virtual patients as “A specific type of computer-based program that simulates real-life clinical scenarios; learners emulate the roles of health care providers to obtain a history, conduct a physical exam, and make diagnostic and therapeutic decisions” [15]. In contrast a definition by Kenny et al. describes VPs as “virtual interactive agents who are trained to simulate a patient’s particular clinical presentation with a high degree of consistency and realism” [16].

There have been a few attempts in recent years to develop a taxonomy for VPs.

While Bearman et al. categorized VPs as either following a problem-solving design (focused on teaching clinical reasoning and diagnosing skills) or a narrative design (emphasis on teaching decision making) [17], Huwendiek et al. have developed a more extensive empirically derived VP typology framework [18] which is based on four categories of metadata (general, educational, instructional and technical) with 19 factors. However, these taxonomies focus on specific types of VPs and suffer from the exclusion of other forms such as high-fidelity simulations or manikins.

A similarly selective view can be seen in systematic reviews such as the one by Cook et al. [19] in which the authors excluded many articles using the virtual patient definition suggested by the AAMC. A more inclusive approach was provided by Talbot et al. in the overview of educational virtual patients identifying seven classes of VPs, ranging from technically basic digital case presentations to advanced virtual reality applications and high-fidelity simulations [20]. This classification takes into account the following features: common names, teaching applications, learner skills evaluated, interactivity, consistency of experience & evaluation, flexibility to recover from learner errors, suitability for game-based approach, author challenge, core, and enabling technology (see Table 1 in http://www.igi-global.com/article/content/74790). However, it is based on experience and does not show the frequency of the terms.

**Table 1:** **Adapted virtual patient classification with two levels of description**

Class label	Predominant competency	Predominant technology	Short description
Case Presentation	Knowledge	Multimedia systems	Interactive multimedia presentation of a patient case to teach primarily basic medical knowledge
Interactive Patient Scenario	Clinical reasoning	Multimedia systems	Interactive multimedia presentation of a patient case to teach mainly clinical reasoning skills (e.g. VPs created for the eViP project)
VP Game	Clinical reasoning or Team training	Virtual worlds	Virtual world to simulate high risk scenarios and team training situations (e.g. Second Life VPs)
High Fidelity Software Simulation	Procedural or basic clinical skills	Dynamic simulations or mixed reality	Real-time simulation of human physiology to teach mainly procedures or skills such as surgical simulations. Non-standard devices (e.g. haptic technology) can be included.
Human Standardized Patient	Patient communication skills	Multimedia systems	Video-recorded actors who role-play a patient to train patient communication skills.
High Fidelity Manikin	Procedural and basic clinical skills, /Team training	Manikins or Part Task Trainers	Manikins with realistic anatomy to train complex procedures such as endoscopy.
Virtual Standardized Patient	Patient communication skills	Conversational characters	A virtual representation of a human being using artificial intelligence technologies and natural language processing to train communication skills.

Additional file 1 shows a more detailed description of the competencies and technologies.

Consequently, the term “virtual patient” is used to describe a multitude of technologies and approaches, making effective communication difficult when educators, researchers and IT specialists share their experiences with VPs.

For example Le Beux and Fieschi used the term “virtual patient” in all three distinguished classes of educational tools: “Simulations for training”, “Virtual reality, video and virtual classroom” and “Medical virtual universities” [21].

Another illustrative example is the article by Huang et al. [22] conducting a survey among US and Canadian medical schools to get an overview about VP activities. One of the results was that 85% of the VPs cost more than $10,000 to develop, but it remained unclear for which kind of VPs this is the case. Costs can vary tremendously between different types of VPs. For example for creating a VP using an authoring tool there are few technical costs compared to programming a high-fidelity VP simulation, which is time-consuming and involves high costs for technical development and equipment.

### Aim

The aim of our project was to investigate and classify variations in the use of the term “virtual patient” with a focus on the healthcare education domain. We also outlined the development of the use of the term over time.

## Methods

### Data extraction

We systematically searched PubMed, Scopus, EMBASE, PsycINFO, CINAHL/EBSCO, and ERIC for citations on virtual patients. Search terms were: “virtual patient” or “virtual patients” in title and/or abstract.

Exclusion criteria were the following:

- article in a language other than English
- short conference abstracts (less than one page)
- term “virtual patient” not mentioned within the article (only in abstract or title)

To ensure a comprehensive search in the literature, we did not use a beginning date cutoff, and the last date of inclusion was December 31st, 2013. All citations were indexed into a spreadsheet including keywords and year of publication. Finally, full-text versions of all included articles were downloaded, read and analyzed.

### General categorization of articles

We categorized the articles based on the proposal by Ellaway [1] as it provided the most comprehensive set of categories. It describes the usage of VPs for educational purposes, electronic patient records and clinical research.

#### Classification of educational articles

We further classified the VPs assigned to the educational category based on the model proposed by Talbot et al. [8]. We chose this framework because it provided the broadest approach as well as the necessary levels of description to classify VPs into the following seven types: Case Presentation, Interactive Patient Scenario, Virtual Patient Game, High Fidelity Software Simulations, Human Standardized Patients, High Fidelity Manikins and Virtual Standardized Patients. Each type is characterized by different descriptive levels such as enabling technology, evaluated learner skills, interactivity or authoring challenge.

Using a deductive content analysis approach [23], we decreased the number of levels in Talbot’s classification to the two most fundamental: educational (“predominant competency”) and technological (“predominant technology”).

Two authors (AK and IH) independently read, categorized and classified each article according to the above described schemata. Conflicts were resolved by consensus with the other authors in case of disagreement. Once all articles were categorized, a descriptive statistical analysis was conducted using Microsoft Excel 2010. No ethical approval was needed, as the study did neither involve any human participants nor any sensitive data.

## Results

**Figure 1:**
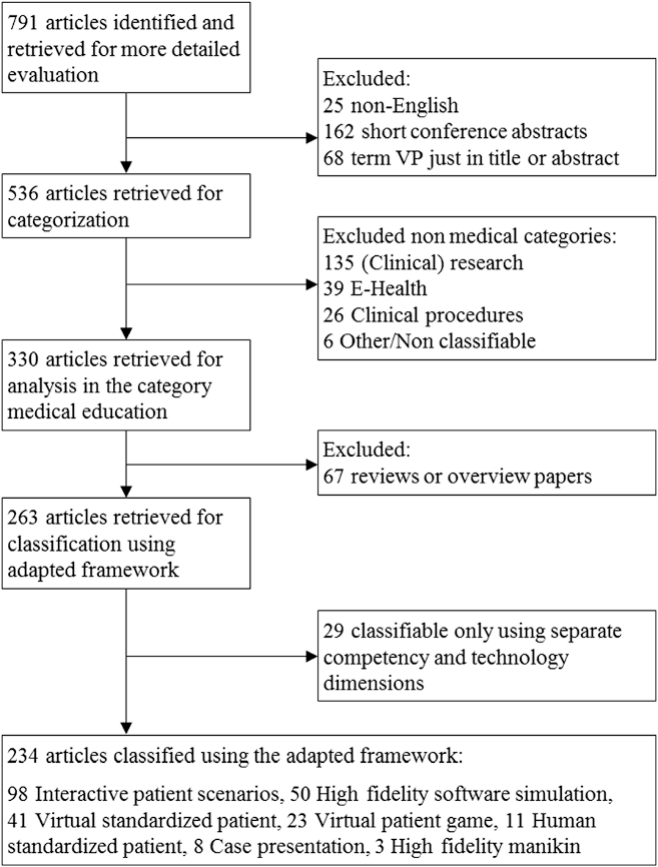
**Progress through the stages of the literature review.**

We identified 791 citations using our search strategy. We excluded 25 non-English articles, 162 short conference abstracts and 68 articles in which the term “virtual patient” was absent in the body of the article. Overall we included 536 articles. The first paper using the term “virtual patient” dated from 1991, the last were published in December 2013. Figure 1 shows the stages of the literature review.

### General categorization of articles

The categories proposed by Ellaway needed further development in order to fit the body of articles. A new category “Clinical Procedures” was introduced under which we subsumed for example computer-assisted surgery (e.g. Kau [24]) or radiotherapy planning (e.g. Guo [25]). In addition we found the “Electronic Patient Records” category too narrow and broadened it to “E-Health” [26], which includes also the use of VPs in the context of telemedicine (e.g. Sicotte et al. [27]) and healthcare information systems (e.g. Effken et al. [28]). Figure 1 shows the number of articles we assigned to each category. Additional file 1 presents a more detailed description of the applied categories.

### Classification of educational articles

Within the largest category, educational articles, 67 (20%) articles were general ones such as reviews or overviews, and therefore classified as “Other”. From the remaining 263 articles, we were able to assign 80% (*n* = 210) to the seven types suggested by Talbot et al. However, we encountered difficulties classifying 20% (*n* = 53) articles for the following reasons:

- Discrepancy of evaluated learner skills and technology level. Talbot’s classification implies by using just one dimension that certain attributes from different levels are bound together. This makes some combinations of attributes across levels impossible (e.g. articles focusing on teaching clinical reasoning but using a high fidelity software simulation, e.g. Kofránek et al. [29]).
- Missing characteristics at particular levels of description - for example we could not classify articles which concerned VPs teaching basic clinical skills (e.g. Lehmann et al. [11]).
- No differentiation - for example the underlying technology of Talbot’s Case Presentation and Interactive Patient Scenario is basically the same (HTML, Authoring tools).

It was not possible to reach consensus for articles having these issues, so we decided to modify the classification and thereby improve the attribution. This modified classification is displayed in Table 1.

**Figure 2:**
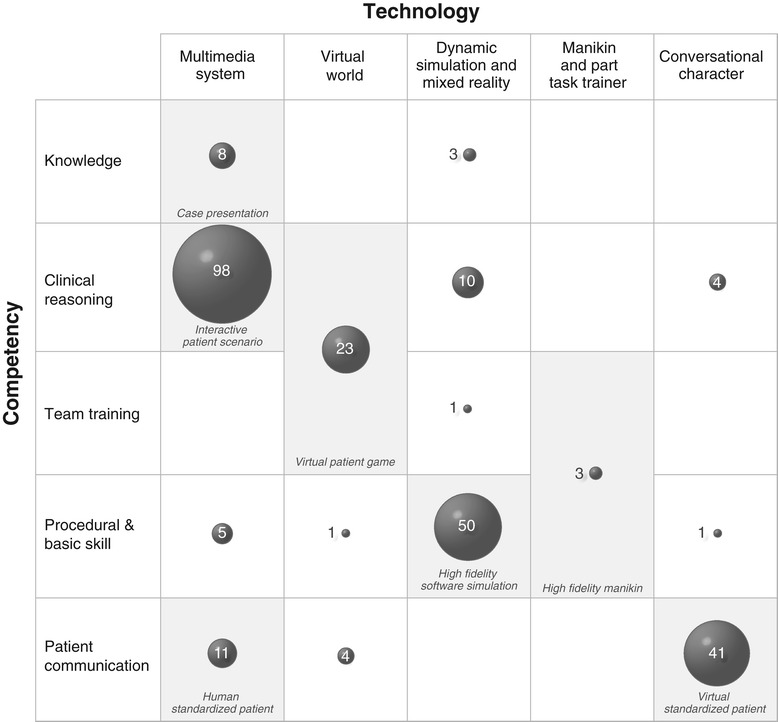
**Overview of VP classes with assigned number of articles.** Grey cells represent the classes of the adapted framework.

By applying this modified one-dimensional classification we were able to classify 234 (89%) of the articles. However, 29 articles could only be assigned to independent technological and competency-based dimensions. Figure 2 shows the distribution of articles based on these two dimensions.

### Development over time

**Figure 3:**
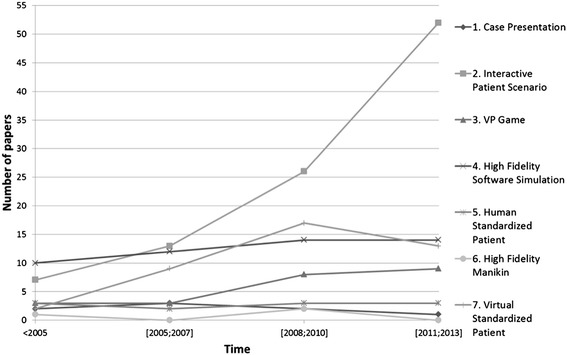
**Development of virtual patients in healthcare education since 1991.**

The first article using the term “virtual patient” in the educational category dates from 1991. It describes a simulation of hemodynamics to teach physiology by Davis et al. [30] Since then the number of published articles using the term “virtual patient” has increased each year, reaching 41 in 2013. Figure 3 shows this development in healthcare education over the last 22 years for the articles assigned to one of the seven classes.

The distribution over the past 20 years of those articles that could not be assigned to one of the seven classes shows no noticeable change, such as increase or decrease.

All articles included in the study are listed in Additional file 2.

## Discussion

### Categorization of VP literature

The categorization of the literature shows that more than 60% of the 536 articles using the term “virtual patient” originate from the healthcare education domain. However, VPs are also quite frequently mentioned in clinical research (25%), but less frequently in clinical procedures (e.g. as surgery planning tools) and as virtual patient records in E-Health. As the focus of our review is education, we did not further analyze the other categories. However, it might be interesting to look into the details of VP usage in these non-educational categories and investigate whether there are concepts or ideas transferable to healthcare education.

### Classification of VP usage in healthcare education articles

When applying our modified classification framework to the body of healthcare education literature most of the articles - 234 (89%) of the 263 - fitted into the modified classification system based on competency and technology. Most (37%) educational articles (excluding “Others”) describe Interactive Patient Scenarios. The next most common classes are VPs in the form of High Fidelity Software Simulations to teach procedures and skills (19%) and Virtual Standardized Patients to train patient communication skills (16%).

In contrast, we only encountered ten (4%) studies using the term “virtual patient” for Human Standardized Patients, eight studies (3%) for Case Presentations and three (1%) for High Fidelity Manikins.

An explanation for these rare occurrences may be that these forms are often not labeled as VPs - for example Case Presentations are also often referred to as Case-based learning (CBL). Our assumption was to include any use of the term “virtual patient” even though there might be more suitable terms (e.g. manikins are usually not called virtual patients). To comprehensively outline articles in these classes - including those describing VPs that are not called VPs within the article - future studies could extend the literature search and include additional search terms (e.g. “patient simulation”), similar to the review done by Cook et al. [19].

Although we were able to classify 234 (89%) articles, we still had 29 (11%) that could not be assigned into our adapted one-dimensional model (see cells with white background in Figure 2).

For example ten articles mainly by Nirenburg et al. describe using dynamic simulations to teach students clinical reasoning (e.g. [31]). Other examples are teaching clinical skills in multimedia systems (e.g. by Germanakis et al. [32]) and conversational characters to teach clinical reasoning (e.g. by Summons et al. [33]).

### Development over time

Looking at the development of VP-related educational articles over time there is an increase in the number of articles about Interactive Patient Scenarios. At the same time the number of articles using VP denoting High Fidelity Simulations and VP Games remained almost constant. This is surprising given the technical possibilities that have advanced rapidly over the past 20 years [34].

However, an explanation for this development could be that the cost of creating virtual patients using for example authoring tools is easier, faster and cheaper than creating high-fidelity applications or virtual worlds [35]. Also by using authoring tools teachers are able to create virtual patients without a team of technicians and instructional designers [36,37]. Further studies could look for cost statements over time and the number of virtual patients created in each classification to verify this assumption.

The increase of Interactive Patient Scenarios is coinciding with the publication of a review article by Cook et al. [38] emphasizing the use of VPs to teach clinical reasoning skills. Moreover the EU-funded project “eViP” (Electronic virtual patients) [35,39,40] published a series of freely available Interactive Patient Scenarios and other resources, such as integration guidelines during the project duration from 2007 until 2010. It is likely that also this project had an influence on how VPs are perceived in the healthcare education community.

The 29 articles mentioning VP types that could not be assigned to one of the classes did not show a clear development over time, which makes it difficult to predict whether these types could be interesting innovations indicating the direction for future VP development and research. However, in case a VP does not fit into one of the classes we recommend that future researcher more explicitly explain which competency they are addressing and on which technology their VP is based on.

### Classification framework

The developed virtual patient classification framework provides a valuable model for educators, VP developers and researchers in healthcare education. It makes the conceptualization of VP use more explicit and supports a more precise communication about the specific type of VP addressed and allows a clearer focus of future research activities.

The use of the framework to classify virtual patients in medical education requires reflection on the predominant competency and technology of the virtual patient. As our research has shown for most of the published research there exists an association between competency and technology (Figure 2). In these cases we recommend to use the class labels as proposed in Table 1. However, we acknowledge that the competency and technology is a continuum. For instance some of the Interactive Patient Scenarios might have incorporated elements from other virtual patient classes (e.g. game-informed elements or outcomes of high fidelity simulations). In such cases it is necessary to consider what is the base technology or main competency.

If no suitable class is present in our framework we propose to be specific in the description which type of predominant technology and competency is present. Further adaptions of the classification might be necessary in the future, especially when new innovative forms of VPs may arise during the next years. An advantage of the adapted classification is that it is easy to present and apply, as it consists of seven classes with only two levels of description. It allows a quick division of virtual patients into substantially different groups. However, we expect that within each class features, methods, outcomes, and VP usage scenarios of the assigned articles may vary significantly, especially for classes containing many articles, such as Interactive Patient Scenario and High Fidelity Simulation. For these types of VP classes specific classification frameworks can be elaborated. For instance the typology developed by Huwendiek et al. [18] describes the variations of Interactive Patient Scenarios in more detail. Such more detailed typologies should be used in addition to our framework whenever available for the reporting of VPs to allow for a better understanding of the used interventions. Future studies could consider the methodological quality of research reports published in individual classes of virtual patients, which has been already investigated for Interactive Patient Scenarios e.g. by Cook et al. [19].

### Limitations

There are potential limitations to our study. The aim of our research was to classify the body of literature about virtual patients. Therefore we focused exclusively on the search term “virtual patient”, not including other potentially related search terms, such as “patient simulation”. Although it would be interesting to include all those articles into the classification, this was not the aim of our study. We are also aware that our approach solely focused on the usage of the term in research activities in healthcare education and might not be entirely transferable to healthcare education in general.

## Conclusion

Our research shows that there are several “communities” using the term “virtual patient” for their activities. Within these communities it might be obvious what is meant by the term, but when communicating and exchanging research results between such communities and to non-VP experts a clear understanding is indispensable. The primary form of VPs in the educational literature are Interactive Patient Scenarios despite rapid technical advances that would nowadays support more complex applications. The adapted classification provides a valuable model for VP developers, educators and researchers in healthcare education to more clearly communicate about the VPs they are using.

## Additional files

Additional file 1:
**Glossary of categories and classes.**
Additional file 2:
**Articles included in the study.**
