# Supplementary material for: Virtual patients - what are we talking about? A framework to classify the meanings of the term in healthcare education
Source: BMC Med Educ. 2015 Feb 1;15:11. doi: 10.1186/s12909-015-0296-3 (PMC4318546; doi:10.1186/s12909-015-0296-3)
Supplement: Additional file 1: — Glossary of categories and classes. [file 12909_2015_296_MOESM1_ESM.docx]

**Glossary of categories and classes**

*Appendix 1 - Table 1: Main categories for the use of the term “virtual patient”*

| **Categories** |  |
| --- | --- |
| Education | Development of competency (knowledge, skills or attitude) of a group of learners in a healthcare related discipline. |
| (Clinical) Research | Contribution to a new development or discovery. Examples include *in-silico* verification of efficiency of treatment methods or medical devices, the development of decision support tools and modelling of the human body or its parts for research purposes. |
| E-Health^[[1]](#footnote-1)^ | Electronic support of healthcare processes such as documentation (electronic health records), healthcare information systems and patient management at distance (telemedicine). |
| (Clinical) Procedure | Medical procedure for a particular patient that represents digitally his/her body (or its parts), e.g. computer-aided surgery (planning, navigation, control). |

*Appendix 1 - Table 2: Description of the competency dimensions of the adapted classification*

| **Competency classes** | Describes within the educational category the type of competency primarily taught or assessed. |
| --- | --- |
| Knowledge | Facts and concepts, which include knowledge from basic sciences, e.g. biochemistry or (patho-)physiology. |
| Clinical reasoning | Application of knowledge to collect and integrate information from various sources to arrive at a diagnosis and management plan^[[2]](#footnote-2)^ |
| Procedural & basic skills | Psychomotor skills such as basic skills (e.g. IV injection, blood pressure measurement) and complex skills (e.g. endoscopic surgery or anaesthesia). Encompasses also more general skills needed to operate medical devices and software. |
| Team training | Skills needed to work in a group, including inter-professional training and practice of surgical or rescue teams. |
| Patient communication skills | Skills enabling professional communication with patients. |

*Appendix 1 - Table 3: Description of the technical dimensions of the adapted classification*

| **Technology classes** | Describes within the educational category the type of technical means to represent the virtual patient. |
| --- | --- |
| Multimedia system | Combination of text, images, audio recordings, animations, video clips presented e.g. in a web-page or CD-ROM application. |
| Virtual World | Computer-generated environment in which the learner is able to move around/interact with this environment as a virtual character (so called avatar) or in a first-person perspective. |
| Dynamic simulation & mixed reality | High fidelity representation of human anatomy or (patho-)physiological processes in the human body. May include the use of additional equipment such as haptic devices. |
| Mannequins and part task trainers | Whole body or its parts (arm, breast) represented physically. The mannequin or part-task trainer may be connected to a computer or include electronic controllers but the focus of the learners is on the physical not digital representation of the patient. |
| Conversational character | Artificial being (represented as a 3D character or via a text chat) which communicates with the learner in natural language. The learner has no representation in the virtual world. |

1. *http://en.wikipedia.org/wiki/EHealth* [↑](#footnote-ref-1)
2. *Cook DA, Triola MM. Virtual patients: a critical literature review and proposed next steps. Med Educ. 2009;43:303–311* [↑](#footnote-ref-2)
