# Supplementary material for: Virtual patients - what are we talking about? A framework to classify the meanings of the term in healthcare education
Source: BMC Med Educ. 2015 Feb 1;15:11. doi: 10.1186/s12909-015-0296-3 (PMC4318546; doi:10.1186/s12909-015-0296-3)
Supplement: Additional file 2: — Articles included in the study. [file 12909_2015_296_MOESM2_ESM.docx]

Articles included in the study

Abbreviations used in the classification column:

1. Case Presentation; 2. Interactive Patient Scenario; 3. VP Game; 4. High Fidelity Software Simulation; 5. Human Standardized Patient 6. High Fidelity Manikin 7. Virtual Standardized Patient

T1&2. Multimedia System; T3. Virtual World; T4. Dynamic Simulation and Mixed Reality; T7. Conversational Character

E1. Knowledge; E2. Clinical Reasoning; E3. Team Training; E4. Procedural Skills; E7. Patient Communication

Other – paper with a general scope such as review or overview.

| **Year** | **Title** | **Authors** | **Journal** | **Classification** |
| --- | --- | --- | --- | --- |
| 1991 | Teaching physiology through simulation of hemodynamics | Davis T, Mark RG | Computers in Cardiology ():649-652 | T4E1 |
| 1995 | Medicine in virtual environments. | Dumay AC | Technol Health Care. 1995 Oct;3(2):75-89. Review. | other |
| 1995 | Navigating physician resources on the Internet. | Ellenberger B | CMAJ. 1995 Apr 15;152(8):1303-7. | other |
| 1997 | Virtual reality: teaching tool of the twenty-first century? | Hoffman H, Vu D | Acad Med. 1997 Dec;72(12):1076-81. | other |
| 1997 | ENT endoscopic surgical training simulator. | Edmond CV Jr, Heskamp D, Sluis D, Stredney D, Sessanna D, Wiet G, Yagel R, Weghorst S, Oppenheimer P, Miller J, Levin M, Rosenberg L. | Stud Health Technol Inform. 1997;39:518-28. | 4 |
| 1997 | A Case-Based Pharmacy Environment: Cognitive Flexibility + Social Constructivism. | Oliver, KM | NN 9--9,1997, presented aEUROCALL 1997 | 2 |
| 1997 | 13. Body, a PC-based anesthesia simulator | Smith NT | Abstracts of the 17th Annual Symposium on Computing in Anesthesia and Intensive Care, International Journal of Clinical Monitoring and Computing 14(2):128-130 | 4 |
| 1998 | Virtual patients for a virtual hospital. | Loke E, Lun KC | Stud Health Technol Inform. 1998;52 Pt 2:1278-81. | 2 |
| 1998 | Using multimedia virtual patients to enhance the clinical curriculum for medical students. | McGee JB, Neill J, Goldman L, Casey E | Stud Health Technol Inform. 1998;52 Pt 2:732-5. | 2 |
| 1998 | SONOSim3D: a multimedia system for sonography simulation and education with an extensible case database. | Ehricke HH | Eur J Ultrasound. 1998 Aug;7(3):225-300. | 4 |
| 1998 | City rounds. The virtual patient. | Nalick, J | USC Health 6 (1): 3 , 1998 | 6 |
| 1998 | Simulation of spinal nerve blocks for training anesthesiology residents | Blezek DJ, Robb RA, Camp JJ, Nauss LA, Martin DP | Proceedings of SPIE - The International Society for Optical Engineering 3262():45-51 | 4 |
| 2000 | Vitreous surgery simulator. | Hikichi T, Yoshida A, Igarashi S, Mukai N, Harada M, Muroi K, Terada T | Arch Ophthalmol. 2000 Dec;118(12):1679-81. | 4 |
| 2000 | Bedside cardiac examination: constancy in a sea of change. | Richardson TR, Moody JM Jr | Curr Probl Cardiol. 2000 Nov;25(11):783-825. Review. | other |
| 2001 | GOLEM--multimedia simulator for medical education. | Kofránek J, Vu LD, Snáselová H, Kerekes R, Velan T | Stud Health Technol Inform. 2001;84(Pt 2):1042-6. | T4E1 |
| 2001 | Comparing student attitudes to different models of the same virtual patient. | Bearman M, Cesnik B | Stud Health Technol Inform. 2001;84(Pt 2):1004-8. | 5 |
| 2001 | Random comparison of 'virtual patient' models in the context of teaching clinical communication skills. | Bearman M, Cesnik B, Liddell M | Med Educ. 2001 Sep;35(9):824-32. | 5 |
| 2001 | Computer assisted learning. A review. | Schittek M, Mattheos N, Lyon HC, Attström R | Eur J Dent Educ. 2001 Aug;5(3):93-100. | other |
| 2001 | A virtual reality patient simulation system for teaching emergency response skills to U.S. Navy medical providers. | Freeman KM, Thompson SF, Allely EB, Sobel AL, Stansfield SA, Pugh WM | Prehosp Disaster Med. 2001 Jan-Mar;16(1):3-8. | 4 |
| 2001 | Time to learn: the outlook for renewal of patient-centred education in the digital age. | Glick TH, Moore GT | Med Educ. 2001 May;35(5):505-9. | other |
| 2001 | One response to a recent challenge from the institute of medicine: A system to credential medical computer skills | Stumpf PG, Ringel S, Quinn MM | Primary Care Update for Ob/Gyns (2001) 8:5 (186-194). Date of Publication: 2001 | T1&2E4 |
| 2001 | Utilization of an integrated interactive virtual patient database in a web-based environment for teaching continuity of care | Clifton Fuhrman Jr. L, Buff WE, Eaddy M, Dollar M | American Journal of Pharmaceutical Education 65(3):271-275 | 2 |
| 2002 | An approach to computer automation of the extracorporeal circulation. | Boschetti F, Mantero S, Miglietta F, Costantino ML, Montevecchi FM, Fumero R | Comput Biol Med. 2002 Mar;32(2):73-83. | 4 |
| 2002 | Revalidation and virtual patients: A vision of the future? | Michell B | In Practice (2002) 24:4 (221-223). Date of Publication: 2002 | other |
| 2003 | A virtual environment for esophageal intubation training. | Kesavadas T, Joshi D, Mayrose J, Chugh K | Stud Health Technol Inform. 2002;85:221-7. | 4 |
| 2003 | Virtual patients help medical students link basic science with clinical care. | Voelker R | JAMA. 2003 Oct 1;290(13):1700-1. No abstract available. | 1 |
| 2003 | The Medical College of Wisconsin's program to strengthen geriatrics education. | Burns E, Bates T, Cohan M, Kowalski K, Olds GR, Simpson D, Duthie EH Jr | WMJ. 2003;102(2):14-7. | 1 |
| 2003 | Is virtual the same as real? Medical students' experiences of a virtual patient. | Bearman M | Acad Med. 2003 May;78(5):538-45. | 5 |
| 2003 | Virtual patient simulator for distributed collaborative medical education. | Caudell TP, Summers KL, Holten J 4th, Hakamata T, Mowafi M, Jacobs J, Lozanoff BK, Lozanoff S, Wilks D, Keep MF, Saiki S, Alverson D. | Anat Rec B New Anat. 2003 Jan;270(1):23-9. | T4E3 |
| 2003 | A virtual patient based on qualitative simulation | Cavazza M., Simo A | International Conference on Intelligent User Interfaces, Proceedings IUI ():19-25 | 3 |
| 2003 | Qualitative Simulation of Shock States in a Virtual Patient | Simo A., Cavazza M | LECTURE NOTES IN COMPUTER SCIENCE (2780):101-111 | 3 |
| 2003 | Virtual tomography: A new approach to efficient human-computer interaction for medical imaging | Teistler M, Bott OJ, Dormeier J, Pretschner DP | Proceedings of SPIE - The International Society for Optical Engineering 5029():512-519 | 4 |
| 2004 | A problem-based e-Learning prototype system for clinical medical education. | Shyu FM, Liang YF, Hsu WT, Luh JJ, Chen HS | Stud Health Technol Inform. 2004;107(Pt 2):983-7. | 2 |
| 2004 | Virtual patients in clinical medicine. | Simo A, Cavazza M, Kijima R | Stud Health Technol Inform. 2004;98:353-9. | 3 |
| 2004 | Virtual patient: a photo-real virtual human for VR-based therapy. | Kiss B, Benedek B, Szijártó G, Csukly G, Simon L, Takács B | Stud Health Technol Inform. 2004;98:154-6. | 7 |
| 2004 | CAMPUS--a flexible, interactive system for web-based, problem-based learning in health care. | Ruderich F, Bauch M, Haag M, Heid J, Leven FJ, Singer R, Geiss HK, Jünger J, Tönshoff B | Stud Health Technol Inform. 2004;107(Pt 2):921-5. | 2 |
| 2004 | Ultrasound training: the virtual patient. | Heer IM, Middendorf K, Müller-Egloff S, Dugas M, Strauss A. | Ultrasound Obstet Gynecol. 2004 Sep;24(4):440-4. | 4 |
| 2004 | Simulation of patient encounters using a virtual patient in periodontology instruction of dental students: design, usability, and learning effect in history-taking skills. | Schittek Janda M, Mattheos N, Nattestad A, Wagner A, Nebel D, Färbom C, Lê DH, Attström R | Eur J Dent Educ. 2004 Aug;8(3):111-9. | 2 |
| 2004 | Training brief intervention with a virtual coach and virtual patients. [References]. | Hayes-Roth B, Amano K, Saker R, Sephton T | Annual Review of CyberTherapy and Telemedicine. Vol.2 2004, pp. 85-95. | 7 |
| 2005 | Virtual patients get real. | Walsh K | Med Educ. 2005 Nov;39(11):1153-4. No abstract available. | other |
| 2005 | Evaluation of an Interactive Case-based Online Network (ICON) in a problem based learning environment. | Nathoo AN, Goldhoff P, Quattrochi JJ | Adv Health Sci Educ Theory Pract. 2005 Aug;10(3):215-30. | 1 |
| 2005 | A new vision for distance learning and continuing medical education. | Harden RM | J Contin Educ Health Prof. 2005 Winter;25(1):43-51. | other |
| 2005 | An integrated environment for plastic surgery support: building virtual patients, simulating interventions, and supporting intraoperative decisions. | Porro I, Schenone A, Fato M, Raposio E, Molinari E, Beltrame F | Comput Med Imaging Graph. 2005 Jul;29(5):385-94. | 4 |
| 2005 | High-tech tools transform the operating room. | Haugh R | Hosp Health Netw. 2005 Jan;79(1):52-6, 2. | other |
| 2005 | Affect in tutoring dialogues. [References]. | Heylen D, Nijholt A, op den Akker R | Applied Artificial Intelligence. Vol.19(3-4), Mar 2005, pp. 287-311. | T7E4 |
| 2006 | Adaptive and longitudinal pharmaceutical care instruction using an interactive voice response/text-to-speech system. | Hussein G, Kawahara N | Am J Pharm Educ. 2006 Apr 15;70(2):37. | T7E2 |
| 2006 | Learning motivational interviewing: scripting a virtual patient. | Villaume WA, Berger BA, Barker BN | Am J Pharm Educ. 2006 Apr 15;70(2):33. | 7 |
| 2006 | Teaching undergraduate nursing students critical thinking: An innovative informatics strategy. | Warren JJ, Connors HR, Weaver C, Simpson R | Stud Health Technol Inform. 2006;122:261-5. | 1 |
| 2006 | Resident physicians' competencies and attitudes in delivering a postnatal diagnosis of Down syndrome. | Ferguson JE 2nd, Kleinert HL, Lunney CA, Campbell LR | Obstet Gynecol. 2006 Oct;108(4):898-905. | 5 |
| 2006 | Radiology education: a glimpse into the future. | Scarsbrook AF, Graham RN, Perriss RW | Clin Radiol. 2006 Aug;61(8):640-8. Review. | other |
| 2006 | The use of virtual patients to teach medical students history taking and communication skills. | Stevens A, Hernandez J, Johnsen K, Dickerson R, Raij A, Harrison C, DiPietro M, Allen B, Ferdig R, Foti S, Jackson J, Shin M, Cendan J, Watson R, Duerson M, Lok B, Cohen M, Wagner P, Lind DS | Am J Surg. 2006 Jun;191(6):806-11. | 7 |
| 2006 | A randomized trial of teaching clinical skills using virtual and live standardized patients. | Triola M, Feldman H, Kalet AL, Zabar S, Kachur EK, Gillespie C, Anderson M, Griesser C, Lipkin M. | J Gen Intern Med. 2006 May;21(5):424-9. | 2 |
| 2006 | Competency in cardiac examination skills in medical students, trainees, physicians, and faculty: a multicenter study. | Vukanovic-Criley JM, Criley S, Warde CM, Boker JR, Guevara-Matheus L,Churchill WH, Nelson WP, Criley JM | Arch Intern Med. 2006 Mar 27;166(6):610-6. Erratum in: Arch Intern Med. 2006 Jun 26;166(12):1294. | 4 |
| 2006 | Development, implementation and pilot evaluation of a Web-based Virtual Patient Case Simulation environment--Web-SP. | Zary N, Johnson G, Boberg J, Fors UG | BMC Med Educ. 2006 Feb 21;6:10. | 2 |
| 2006 | Interactive educational diabetes/insulin tutorial at www.2aida.info. | Reed K, Lehmann ED | Diabetes Technol Ther. 2006 Feb;8(1):126-37. | T4E1 |
| 2006 | Intelligent inferencing and haptic simulation for Chinese acupuncture learning and training. | Heng PA, Wong TT, Yang R, Chui YP, Xie YM, Leung KS, Leung PC | IEEE Trans Inf Technol Biomed. 2006 Jan;10(1):28-41. | 4 |
| 2006 | Virtual patients: assessment of synthesized versus recorded speech. | Dickerson R, Johnsen K, Raij A, Lok B, Stevens A, Bernard T, Lind DS | Stud Health Technol Inform. 2006;119:114-9. | 7 |
| 2006 | Virtual reality simulation training can improve inexperienced surgeons' endovascular skills. | Aggarwal R, Black SA, Hance JR, Darzi A, Cheshire NJ | Eur J Vasc Endovasc Surg. 2006 Jun;31(6):588-93. Epub 2006 Jan 4. | 4 |
| 2006 | The Virtual Patient"--Development, Implementation and Evaluation of an Innovative Computer Simulation for Postgraduate Nursing Students | Kiegaldie D, White G | Journal of Educational Multimedia and Hypermedia,15,1,31--47,2006 | T1&2E4 |
| 2006 | Immersive open surgery simulation | Al-khalifah A, McCrindle R, Alexandrov V | Lecture Notes in Computer Science (including subseries Lecture Notes in Artificial Intelligence and Lecture Notes in Bioinformatics) 2006 3991 LNCS - I():868-871 | 4 |
| 2006 | Searching for a specific virtual environment for an angioplasty training simulator | Aloisio G, De Paolis LT, Provenzano L | WSEAS Transactions on Information Science and Applications 3(4):720-728 | 4 |
| 2006 | The virtual movable human upper body for palpatory diagnostic training | Chen MY, Williams II RL, Conatser Jr. RR, Howell JN | SAE Technical Papers ():- | 4 |
| 2006 | A haptic needle manipulation simulator for chinese acupuncture learning and training | Heng PA, Wong TT, Leung KM, Chui YP, Sun H | Proceedings VRCAI 2004 - ACM SIGGRAPH International Conference on Virtual Reality Continuum and its Applications in Industry ():57-64 | 4 |
| 2006 | Teaching communication skills with virtual humans | Lok B | IEEE Computer Graphics and Applications 26(3):10-13 | 7 |
| 2006 | Applying virtual reality in medical communication education: Current findings and potential teaching and learning benefits of immersive virtual patients | Lok B, Ferdig RE, Raij A, Johnsen K, Dickerson R, Coutts J, Stevens A, Lind DS | Virtual Reality 10(41337):185-195 | 7 |
| 2006 | Cognitive simulation in Virtual Patients | Nirenburg S., McShane M., Beale S., O'Hara T., Jarrell B., Fantry G., Raczek J. | FLAIRS 2006 - Proceedings of the Nineteenth International Florida Artificial Intelligence Research Society Conference 2006():174-175 | T4E2 |
| 2006 | A hybrid virtual environment for training of radiotherapy treatment of cancer | Phillips R, Ward JW, Bridge P, Appleyard RM, Beavis AW | Proceedings of SPIE - The International Society for Optical Engineering 6055():- | 4 |
| 2006 | Interpersonal scenarios: Virtual ≈ real? | Raij A, Johnsen K, Dickerson R, Lok B, Cohen M, Stevens A, Bernard T, Oxendine C, Wagner P, Scott Lind D | Proceedings - IEEE Virtual Reality 2006():8- | 7 |
| 2006 | J-Ortho: An open-source orthodontic treatment simulator | Rodrigues M.A.F., Ribeiro I.M.M.P., Silva W.B., Barbosa R.G., Neto M.E.B. | Proceedings of the ACM Symposium on Applied Computing 1():245-249 | 4 |
| 2006 | Evaluation of an online analogical patient simulation program | Thompson GA, Holyoak KJ, Morrison RG, Clark TK | Proceedings - IEEE Symposium on Computer-Based Medical Systems 2006():623-628 | 2 |
| 2007 | An XML standard for virtual patients: exchanging case-based simulations in medical education. | Triola MM, Campion N, McGee JB, Albright S, Greene P, Smothers V, Ellaway R | AMIA Annu Symp Proc. 2007 Oct 11:741-5. | other |
| 2007 | Modeling relief. | Sumner W 2nd, Xu JZ, Roussel G, Hagen MD. | AMIA Annu Symp Proc. 2007 Oct 11:706-10. | T4E2 |
| 2007 | Virtual patient model for multi-person virtual medical environments. | Dev P, Heinrichs WL, Youngblood P, Kung S, Cheng R, Kusumoto L, Hendrick A | AMIA Annu Symp Proc. 2007 Oct 11:181-5. | 3 |
| 2007 | Caring for children with intellectual and developmental disabilities: virtual patient instruction improves students' knowledge and comfort level. | Sanders CL, Kleinert HL, Free T, Slusher I, Clevenger K, Johnson S, Boyd SE. | J Pediatr Nurs. 2007 Dec;22(6):457-66. | 2 |
| 2007 | Patient-centred learning--back to the future. | Smith SR, Cookson J, McKendree J, Harden RM | Med Teach. 2007 Feb;29(1):33-7. | other |
| 2007 | Integrating virtual patients into a self-care course. | Orr KK | Am J Pharm Educ. 2007 Apr 15;71(2):30. | 5 |
| 2007 | Do medical students respond empathetically to a virtual patient? | Deladisma AM, Cohen M, Stevens A, Wagner P, Lok B, Bernard T, Oxendine C, Schumacher L, Johnsen K, Dickerson R, Raij A, Wells R, Duerson M, Harper JG, Lind DS; Association for Surgical Education | Am J Surg. 2007 Jun;193(6):756-60. | 7 |
| 2007 | Virtual patient simulation at US and Canadian medical schools. | Huang G, Reynolds R, Candler C | Acad Med. 2007 May;82(5):446-51. | other |
| 2007 | Virtual biomedical universities and e-learning. | Beux PL, Fieschi M | Int J Med Inform. 2007 May-Jun;76(5-6):331-5. Review. | other |
| 2007 | A serious gaming/immersion environment to teach clinical cancer genetics. | Nosek TM, Cohen M, Matthews A, Papp K, Wolf N, Wrenn G, Sher A, Coulter K, Martin J, Wiesner GL | Stud Health Technol Inform. 2007;125:355-60. | 1 |
| 2007 | An interactive, cognitive simulation of gastroesophageal reflux disease. | Jarrell B, Nirenburg S, McShane M, Fantry G, Beale S, Mallott D, Raczek J | Stud Health Technol Inform. 2007;125:194-9. | T4E2 |
| 2007 | Localized virtual patient model for regional anesthesia simulation training system. | Hu J, Lim YJ, Tardella N, Chang C, Warren L | Stud Health Technol Inform. 2007;125:185-90. | 4 |
| 2007 | Training inter-physician communication using the Dynamic Patient Simulator. | Sijstermans R, Jaspers MW, Bloemendaal PM, Schoonderwaldt EM | Int J Med Inform. 2007 May-Jun;76(5-6):336-43. Epub 2007 Feb 28. | 2 |
| 2007 | Improving student dentist competencies and perception of difficulty in delivering care to children with developmental disabilities using a virtual patient module. | Kleinert HL, Sanders C, Mink J, Nash D, Johnson J, Boyd S, Challman S | J Dent Educ. 2007 Feb;71(2):279-86. | 2 |
| 2007 | Virtual patients in undergraduate surgery education: a randomized controlled study. | Vash JH, Yunesian M, Shariati M, Keshvari A, Harirchi I | ANZ J Surg. 2007 Jan-Feb;77(1-2):54-9. | 2 |
| 2007 | Transforming Professional Healthcare Narratives into Structured Game-Informed-Learning Activities | Begg M, Ellaway R, Dewhurst D, Macleod H | Innovate: Journal of Online Education",3,6,7--7,2007 | other |
| 2007 | The Development and Evaluation of a Virtual Radiotherapy Treatment Machine Using an Immersive Visualisation Environment | Bridge P, Appleyard MR, Ward,WJ, Philips R, Beavis WA | Computers & Education,49,2,481--494,2007 | 4 |
| 2007 | Innovative Technologies for Multicultural Education Needs | Ferdig RE, Coutts J, DiPietro J, Lok B, Davis N | Multicultural Education & Technology Journal,1,1,47--63,2007 | 7 |
| 2007 | Clinical Reasoning Skills of Speech and Language Therapy Students | Hoben K, Varley R, Cox R | International Journal of Language & Communication Disorders,42,13--13,2007 | 2 |
| 2007 | Improving physician assistants students' competencies in developmental disabilities using virtual patient modules. | Kleinert, HL, Fisher SB, Sanders CL,Boyd S | Journal of Physician Assistant Education, 18(2): 33-40, 2007 | 2 |
| 2007 | Use and evaluation of "virtual" patients for assessment of clinical pharmacy undergraduates. [References]. | Marriott JL | Pharmacy Education: An International Journal of Pharmaceutical Education. Vol.7(4), 2007, pp. 341-349. | 2 |
| 2007 | Development and implementation of a computer-generated "virtual" patient program. [References]. | Marriott JL | Pharmacy Education: An International Journal of Pharmaceutical Education. Vol.7(4), 2007, pp. 335-340. | 2 |
| 2007 | Teaching decision-making skills through inexpensive virtual scenarios | Conradi E, Poulton T, Round J | Proceedings of the 10th IASTED International Conference on Computers and Advanced Technology in Education ():404-409 | 2 |
| 2007 | Vicarious learning and (virtual) case-based teaching in health science education | Cox R, Pang J | Proceedings - IEEE Symposium on Computer-Based Medical Systems , art. no. 4262723 , pp. 657-662 | 2 |
| 2007 | A distributed virtual reality-based system for neonatal decision-making training | Holobar A, Divjak M, Prelog I, Korosec D, Zazula D | Computer Applications in Engineering Education 15(4):329-339 | 3 |
| 2007 | Virtual patients for clinical therapist skills training | Kenny P, Parsons TD, Gratch J, Leuski A, Rizzo AA | Lecture Notes in Computer Science (including subseries Lecture Notes in Artificial Intelligence and Lecture Notes in Bioinformatics) 4722 LNCS():197-210 | 7 |
| 2007 | Knowledge-based modeling and simulation of diseases with highly differentiated clinical manifestations | McShane M, Nirenburg S, Beale S, Jarrell B, Fantry G | Lecture Notes in Computer Science (including subseries Lecture Notes in Artificial Intelligence and Lecture Notes in Bioinformatics) 4594 LNAI():34-43 | T4E2 |
| 2007 | Medical education interfaces through virtual patients based on qualitative simulation | Simo A, Cavazza M | Studies in Computational Intelligence 48():255-290 | 3 |
| 2008 | Virtual patient simulation for prevention of medical error: beyond just technical upskilling. | McConnell H, Pardy A | World Hosp Health Serv. 2008;44(3):36-9. | other |
| 2008 | High degree of realism in teaching percutaneous coronary interventions by combining a virtual reality trainer with a full scale patient simulator. | Schuetz M, Moenk S, Vollmer J, Kurz S, Mollnau H, Post F, Heinrichs W | Simul Healthc. 2008 Winter;3(4):242-6. doi: 10.1097/SIH.0b013e3181871b58. | 6 |
| 2008 | The development of a competency-based group health teaching performance examination model for BSN graduates. | Tai CY, Chung UL | J Nurs Res. 2008 Dec;16(4):275-85. | 5 |
| 2008 | Virtual patients and undergraduate anaesthesia teaching. | Critchley LA, Wong JW, Leung JY | Med Educ. 2008 Nov;42(11):1120-1. doi: 10.1111/j.1365-2923.2008.03194.x. No abstract available. | 2 |
| 2008 | Virtual standardized patients: an interactive method to examine variation in depression care among primary care physicians. | Hooper LM, Weinfurt KP, Cooper LA, Mensh J, Harless W, Kuhajda MC, Epstein SA | Prim Health Care Res Dev. 2008 Oct 1;9(4):257-268. | 5 |
| 2008 | Virtual patient instruction for dental students: can it improve dental care access for persons with special needs? | Sanders C, Kleinert HL, Boyd SE, Herren C, Theiss L, Mink J. | Spec Care Dentist. 2008 Sep-Oct;28(5):205-13. doi: 10.1111/j.1754-4505.2008.00038.x. | 2 |
| 2008 | Virtual patient training to improve reproductive health care for women with intellectual disabilities. | Boyd SE, Sanders CL, Kleinert HL, Huff MB, Lock S, Johnson S, Clevenger K, Bush NA, Van Dyke E, Clark TL | J Midwifery Womens Health. 2008 Sep-Oct;53(5):453-60. doi: 10.1016/j.jmwh.2008.04.017. | 2 |
| 2008 | Using virtual patients to improve cardiac examination competency in medical students. | Vukanovic-Criley JM, Boker JR, Criley SR, Rajagopalan S, Criley JM | Clin Cardiol. 2008 Jul;31(7):334-9. doi: 10.1002/clc.20213. | 4 |
| 2008 | The use of a virtual patient case in an OSCE-based exam--a pilot study. | Courteille O, Bergin R, Stockeld D, Ponzer S, Fors U | Med Teach. 2008;30(3):e66-76. doi: 10.1080/01421590801910216. | 2 |
| 2008 | Building a virtual patient commons. | Ellaway R, Poulton T, Fors U, McGee JB, Albright S | Med Teach. 2008;30(2):170-4. doi: 10.1080/01421590701874074. | other |
| 2008 | Virtual reality training for radiotherapy becomes a reality. | Phillips R, Ward JW, Page L, Grau C, Bojen A, Hall J, Nielsen K, Nordentoft V, Beavis AW | Stud Health Technol Inform. 2008;132:366-71. | 4 |
| 2008 | Objective structured clinical interview training using a virtual human patient. | Parsons TD, Kenny P, Ntuen CA, Pataki CS, Pato MT, Rizzo AA, St-George C, Sugar J | Stud Health Technol Inform. 2008;132:357-62. | 7 |
| 2008 | Revealing the conceptual substrate of biomedical cognitive models to the wider community. | McShane M, Jarrell B, Fantry G, Nirenburg S, Beale S, Johnson B | Stud Health Technol Inform. 2008;132:281-6. | other |
| 2008 | Design and implementation of rule-based medical models: an In Silico patho-physiological trauma model for hypovolemic shock. | Heinrichs WL, Kung SY, Dev P | Stud Health Technol Inform. 2008;132:159-64. | 3 |
| 2008 | Medical student satisfaction using a virtual patient system to learn history-taking communication skills. | Deladisma AM, Johnsen K, Raij A, Rossen B, Kotranza A, Kalapurakal M, Szlam S, Bittner JG 4th, Swinson D, Lok B, Lind DS | Stud Health Technol Inform. 2008;132:101-5. | 7 |
| 2008 | Developmental disabilities: improving competence in care using virtual patients. | Sanders CL, Kleinert HL, Free T, King P, Slusher I, Boyd S | J Nurs Educ. 2008 Feb;47(2):66-73. | 2 |
| 2008 | Virtual patients for assessing medical students--important aspects when considering the introduction of a new assessment format. | Waldmann UM, Gulich MS, Zeitler HP | Med Teach. 2008 Feb;30(1):17-24. doi: 10.1080/01421590701758616. | 2 |
| 2008 | E-learning virtual patients for geratric education. | Orton E, Mulhausen P | Gerontol Geriatr Educ. 2008;28(3):73-88. doi: 10.1300/J021v28n03_06. | 2 |
| 2008 | Can Virtual Patients Help Real Professors Teach Medicine? | Debolt D | Chronicle of Higher Education,55,16,1--A6,2008 | T7E2 |
| 2008 | Game-informed learning and teaching in healthcare education. | Begg M | Health Information on the Internet 66: 8-9, 2008 | other |
| 2008 | SAILOR: A 3-D medical simulator of Loco-Regional Anaesthesia based on desktop virtual reality and pseudo-haptic feedback | Bibin L, Lecuyer A, Burkhardt JM, Bonnet M, Delbos A | Proceedings of the ACM Symposium on Virtual Reality Software and Technology, VRST ():97-100 | 4 |
| 2008 | Learning medicine through collaboration and action: Collaborative, experiential, networked learning environments | Dev P, Heinrichs Wm LR | Virtual Reality 12(4):215-234 | 3 |
| 2008 | Virtual intelligent agents to train abilities of diagnosis in psychology and psychiatry | Gutierrez-Maldonado J, Alsina-Jurnet I, Rangel-Gomez MV, Aguilar-Alonso A, Jarne-Esparcia AJ, Andres-Pueyo A, Talarn-Caparros A | Studies in Computational Intelligence 142():497-505 | 7 |
| 2008 | The virtual patient project: Using low fidelity, student generated online cases in medical education | Imison M., Hughes C | ASCILITE 2008 - The Australasian Society for Computers in Learning in Tertiary Education ():441-445 | 2 |
| 2008 | Virtual humans for assisted health care | Kenny P, Parsons T, Gratch J, Rizzo A | 1st International Conference on Pervasive Technologies Related to Assistive Environments, PETRA 2008 ():- | 7 |
| 2008 | Evaluation of Justina: A virtual patient with PTSD | Kenny P, Parsons TD, Gratch J, Rizzo AA | Lecture Notes in Computer Science (including subseries Lecture Notes in Artificial Intelligence and Lecture Notes in Bioinformatics) 5208 LNAI():394-408 | 7 |
| 2008 | Virtual human + tangible interface = Mixed reality human an initial exploration with a virtual breast exam patient | Kotranza A, Lok B | Proceedings - IEEE Virtual Reality ():99-106 | 6 |
| 2008 | A tool for training primary health care medical students: The virtual simulated patient | Lopez V, Eisman EM, Castro JL | Proceedings - International Conference on Tools with Artificial Intelligence, ICTAI 2():194-201 | 7 |
| 2008 | A simulated physiological/cognitive "Double Agent" | Nirenburg S, McShane M, Beale S | AAAI Fall Symposium - Technical Report FS-08-04():127-134 | T4E2 |
| 2008 | Designing game-based learning activities for virtual patients in second life | Toro-Troconis M., Mellstrom U., Partridge M., Meeran K., Barrett M., Higham J | Journal of Cyber Therapy and Rehabilitation 1(3):225-238 | 3 |
| 2009 | Virtual patient simulator for the perfusion resource management drill. | Ninomiya S, Tokaji M, Tokumine A, Kurosaki T | J Extra Corpor Technol. 2009 Dec;41(4):206-12. | 4 |
| 2009 | An intersubject variable regional anesthesia simulator with a virtual patient architecture. | Ullrich S, Grottke O, Fried E, Frommen T, Liao W, Rossaint R, Kuhlen T, Deserno TM | Int J Comput Assist Radiol Surg. 2009 Nov;4(6):561-70. doi: 10.1007/s11548-009-0371-5. Epub 2009 Jun 13. | 4 |
| 2009 | Blended E-learning in a Web-based virtual hospital: a useful tool for undergraduate education in urology. | Horstmann M, Renninger M, Hennenlotter J, Horstmann CC, Stenzl A | Educ Health (Abingdon). 2009 Aug;22(2):269. Epub 2009 Jul 30. | 2 |
| 2009 | Second life for dental education. | Phillips J, Berge ZL | J Dent Educ. 2009 Nov;73(11):1260-4. | 3 |
| 2009 | Virtual patient safety rounds: one hospital system's approach to sharing knowledge. | Graham JM, Brinson M, Magtibay LV, Regan B, Lazar EJ | J Healthc Qual. 2009 Sep-Oct;31(5):48-52. | other |
| 2009 | Training staff to create simple interactive virtual patients: the impact on a medical and healthcare institution. | Round J, Conradi E, Poulton T | Med Teach. 2009 Aug;31(8):764-9. | other |
| 2009 | Improving assessment with virtual patients. | Round J, Conradi E, Poulton T | Med Teach. 2009 Aug;31(8):759-63. | other |
| 2009 | The replacement of 'paper' cases by interactive online virtual patients in problem-based learning. | Poulton T, Conradi E, Kavia S, Round J, Hilton S | Med Teach. 2009 Aug;31(8):752-8. | 2 |
| 2009 | Creation of virtual patients from CT images of cadavers to enhance integration of clinical and basic science student learning in anatomy. | Jacobson S, Epstein SK, Albright S, Ochieng J, Griffiths J, Coppersmith V, Polak JF | Med Teach. 2009 Aug;31(8):749-51. | 1 |
| 2009 | Towards a typology of virtual patients. | Huwendiek S, De leng BA, Zary N, Fischer MR, Ruiz JG, Ellaway R | Med Teach. 2009 Aug;31(8):743-8. | other |
| 2009 | The use of virtual patients to assess the clinical skills and reasoning of medical students: initial insights on student acceptance. | Gesundheit N, Brutlag P, Youngblood P, Gunning WT, Zary N, Fors U | Med Teach. 2009 Aug;31(8):739-42. | 2 |
| 2009 | Cross-cultural use and development of virtual patients. | Fors UG, Muntean V, Botezatu M, Zary N | Med Teach. 2009 Aug;31(8):732-8. | 2 |
| 2009 | Online virtual patients - A driver for change in medical and healthcare professional education in developing countries? | Dewhurst D, Borgstein E, Grant ME, Begg M | Med Teach. 2009 Aug;31(8):721-4. | 2 |
| 2009 | Virtual patients in a virtual world: Training paramedic students for practice. | Conradi E, Kavia S, Burden D, Rice A, Woodham L, Beaumont C, Savin-Baden M, Poulton T | Med Teach. 2009 Aug;31(8):713-20. | 3 |
| 2009 | The role of intellectual property in creating, sharing and repurposing virtual patients. | Campbell G, Miller A, Balasubramaniam C | Med Teach. 2009 Aug;31(8):709-12. | other |
| 2009 | Virtual patients come of age. | Ellaway RH, Poulton T, Smothers V, Greene P | Med Teach. 2009 Aug;31(8):683-4. | other |
| 2009 | Medical education in Korea: the e-learning consortium. | Kim KJ, Han J, Park IeB, Kee C | Med Teach. 2009 Sep;31(9):e397-401. Review. | other |
| 2009 | Enabling interoperability, accessibility and reusability of virtual patients across Europe - design and implementation. | Zary N, Hege I, Heid J, Woodham L, Donkers J, Kononowicz AA | Stud Health Technol Inform. 2009;150:826-30. | other |
| 2009 | Development and validation of strategies to test for interoperability of virtual patients. | Kononowicz AA, Heid J, Donkers J, Hege I, Woodham L, Zary N | Stud Health Technol Inform. 2009;150:185-9. | other |
| 2009 | Integration of patient-specific paranasal sinus computed tomographic data into a virtual surgical environment. | Parikh SS, Chan S, Agrawal SK, Hwang PH, Salisbury CM, Rafii BY, Varma G, Salisbury KJ, Blevins NH | Am J Rhinol Allergy. 2009 Jul-Aug;23(4):442-7. doi: 10.2500/ajra.2009.23.3335. | 4 |
| 2009 | Improving residents' understanding of issues, comfort levels, and patient needs regarding screening for and diagnosing Down syndrome. | Kleinert HL, Lunney CA, Campbell L, Ferguson JE 2nd | Am J Obstet Gynecol. 2009 Sep;201(3):328.e1-6. doi: 10.1016/j.ajog.2009.05.051. Epub 2009 Jul 24. | 5 |
| 2009 | Optimizing Clinical Training for the Treatment of PTSD Using Virtual Patients. | Kenny PG, Parsons TD, Rothbaum B, Difede J, Reger G, Rizzo A | Stud Health Technol Inform. 2009;144:264-8. | 7 |
| 2009 | A Comparative Analysis between Experts and Novices Interacting with a Virtual Patient with PTSD. | Kenny PG, Parsons TD, Rizzo A | Stud Health Technol Inform. 2009;144:122-4. | 7 |
| 2009 | Integration strategies for using virtual patients in clinical clerkships. | Berman N, Fall LH, Smith S, Levine DA, Maloney CG, Potts M, Siegel B, Foster-Johnson L | Acad Med. 2009 Jul;84(7):942-9. doi: 10.1097/ACM.0b013e3181a8c668. | 2 |
| 2009 | 12 Tips: Guidelines for authoring virtual patient cases. | Posel N, Fleiszer D, Shore BM | Med Teach. 2009 Aug;31(8):701-8. doi: 10.1080/01421590902793867. | other |
| 2009 | Design principles for virtual patients: a focus group study among students. | Huwendiek S, Reichert F, Bosse HM, de Leng BA, van der Vleuten CP, Haag M, Hoffmann GF, Tönshoff B | Med Educ. 2009 Jun;43(6):580-8. doi: 10.1111/j.1365-2923.2009.03369.x. | 2 |
| 2009 | The use of virtual reality simulation of head trauma in a surgical boot camp. | Vergara VM, Panaiotis, Kingsley D, Alverson DC, Godsmith T, Xia S, Caudell TP | Stud Health Technol Inform. 2009;142:395-7. | 4 |
| 2009 | Integrating cognitive simulation into the Maryland virtual patient. | Nirenburg S, McShane M, Beale S, Jarrell B, Fantry G | Stud Health Technol Inform. 2009;142:224-9. | 4 |
| 2009 | Virtual patients: a critical literature review and proposed next steps. | Cook DA, Triola MM | Med Educ. 2009 Apr;43(4):303-11. doi: 10.1111/j.1365-2923.2008.03286.x. Review. | other |
| 2009 | Interactive Internet-based cases for undergraduate otolaryngology education. | Kandasamy T, Fung K | Otolaryngol Head Neck Surg. 2009 Mar;140(3):398-402. doi: 10.1016/j.otohns.2008.11.033. | 2 |
| 2009 | Web-based virtual patients in dentistry: factors influencing the use of cases in the Web-SP system. | Zary N, Johnson G, Fors U | Eur J Dent Educ. 2009 Feb;13(1):2-9. doi: 10.1111/j.1600-0579.2007.00470.x. | 2 |
| 2009 | A pilot study to integrate an immersive virtual patient with a breast complaint and breast examination simulator into a surgery clerkship. | Deladisma AM, Gupta M, Kotranza A, Bittner JG 4th, Imam T, Swinson D, Gucwa A, Nesbit R, Lok B, Pugh C, Lind DS | Am J Surg. 2009 Jan;197(1):102-6. doi: 10.1016/j.amjsurg.2008.08.012. | 7 |
| 2009 | Knowledge-Driven Design of Virtual Patient Simulations | Vergara V, Caudell T, Goldsmith T, Alverson D | Innovate: Journal of Online Education,5,2,,6--6,2009 | 3 |
| 2009 | A novel case-based approach to continuing medical education using a virtual patient record. | Jones PE, Mulitalo KE | Journal of Physician Assistant Education, 20(3):28-30, 2009 | 2 |
| 2009 | mEducator: A best practice network for repurposing and sharing medical educational multi-type content | Bamidis PD, Kaldoudi E, Pattichis C | IFIP Advances in Information and Communication Technology 307():769-776 | other |
| 2009 | The virtual haptic human upper body | Chen MY, Williams II RL | Proceedings of the ASME International Design Engineering Technical Conferences and Computers and Information in Engineering Conference 2009, DETC2009 7(PART B):1079-1087 | 4 |
| 2009 | A framework for visuo-haptic simulation of puncture interventions | Faerber M, Dalek D, Habermann CR, Hummel F, Schops C, Handels H | INFORMATIK 2009 - Im Focus das Leben, Beitrage der 39. Jahrestagung der Gesellschaft fur Informatik e.V. (GI) ():1309-1316 | 4 |
| 2009 | Efficacy of active participation in conversation with a virtual patient with Alzheimer's disease | Green NL, Bevan C | AAAI Fall Symposium - Technical Report FS-09-07():15-19 | 7 |
| 2009 | Using virtual patients to train clinical interviewing skills | Hayes-Roth B, Saker R, Amano K | AAAI Fall Symposium - Technical Report FS-09-07():35-40 | 7 |
| 2009 | Integrating conversational virtual humans and mannequin patient simulators to present mixed reality clinical training experiences | Hwang Y, Lampotang S, Gravenstein N, Luria I, Lok B | Science and Technology Proceedings - IEEE 2009 International Symposium on Mixed and Augmented Reality, ISMAR 2009 ():197-198 | 7 |
| 2009 | Evaluation of novice and expert interpersonal interaction skills with a virtual patient | Kenny PG, Parsons TD, Gratch J, Rizzo AA | Lecture Notes in Computer Science (including subseries Lecture Notes in Artificial Intelligence and Lecture Notes in Bioinformatics) 5773 LNAI():511-512 | 7 |
| 2009 | Human computer interaction in virtual standardized patient systems | Kenny PG, Parsons TD, Rizzo AA | Lecture Notes in Computer Science (including subseries Lecture Notes in Artificial Intelligence and Lecture Notes in Bioinformatics) 5613 LNCS(PART 4):514-523 | 7 |
| 2009 | Reducing the effort in the creation of new patients using the virtual simulated patient framework | Lopez Salazar V, Castro Pena JL, Vazquez Granado J | IEEE International Conference on Fuzzy Systems ():764-769 | 7 |
| 2009 | Development and evaluation of a virtual reality patient simulation (VRPS) | Nestler S, Huber M, Echtler F, Dollinger A, Klinker G | 17th International Conference in Central Europe on Computer Graphics, Visualization and Computer Vision, WSCG'2009 - In Co-operation with EUROGRAPHICS, Full Papers Proceedings ():169-175 | 4 |
| 2009 | Cognitive modeling for clinical medicine | Nirenburg S, McShane M | AAAI Fall Symposium - Technical Report FS-09-07():52-59 | T4E2 |
| 2009 | Design strategy for a scalable virtual pharmacy patient | Summons PF, Newby D, Athauda R, Park M, Shaw P, Pranata I, Jin JS, Xu YD | ACIS 2009 Proceedings - 20th Australasian Conference on Information Systems ():96-110 | T7E2 |
| 2010 | Randomized, controlled trial of a normal pregnancy virtual patient to teach medical students counseling skills. | Ricciotti HA, Hacker MR, De Flesco LD, Dodge LE, Huang GC | J Reprod Med. 2010 Nov-Dec;55(11-12):498-502. | 2 |
| 2010 | Virtual patients and problem-based learning in advanced therapeutics. | Benedict N | Am J Pharm Educ. 2010 Oct 11;74(8):143. | 2 |
| 2010 | Virtual patients: practical advice for clinical authors using Labyrinth. | Begg M | Clin Teach. 2010 Sep;7(3):202-5. doi: 10.1111/j.1743-498X.2010.00382.x. | other |
| 2010 | Virtual patient simulation: what do students make of it? A focus group study. | Botezatu M, Hult H, Fors UG | BMC Med Educ. 2010 Dec 4;10:91. doi: 10.1186/1472-6920-10-91. | 2 |
| 2010 | Training healthcare personnel for mass-casualty incidents in a virtual emergency department: VED II. | Heinrichs WL, Youngblood P, Harter P, Kusumoto L, Dev P | Prehosp Disaster Med. 2010 Sep-Oct;25(5):424-32. | 3 |
| 2010 | As time goes by: Stakeholder opinions on the implementation and use of a virtual patient simulation system. | Botezatu M, Hult H, Kassaye Tessma M, Fors UG | Med Teach. 2010;32(11):e509-16. doi: 10.3109/0142159X.2010.519066. | other |
| 2010 | Review: women's health module. | Callahan KE | J Am Geriatr Soc. 2010 Sep;58(9):1786-7. doi: 10.1111/j.1532-5415.2010.03027.x. Review. | 2 |
| 2010 | Virtual patient simulation for learning and assessment: Superior results in comparison with regular course exams. | Botezatu M, Hult H, Tessma MK, Fors UG | Med Teach. 2010;32(10):845-50. doi: 10.3109/01421591003695287. | 2 |
| 2010 | Using a web-based orthopaedic clinic in the curricular teaching of a German university hospital: analysis of learning effect, student usage and reception. | Wünschel M, Leichtle U, Wülker N, Kluba T | Int J Med Inform. 2010 Oct;79(10):716-21. doi: 10.1016/j.ijmedinf.2010.07.007. Epub 2010 Aug 21. | 2 |
| 2010 | Computerized virtual patients in health professions education: a systematic review and meta-analysis. | Cook DA, Erwin PJ, Triola MM | Acad Med. 2010 Oct;85(10):1589-602. doi: 10.1097/ACM.0b013e3181edfe13. Review. | other |
| 2010 | Virtual reality triage training provides a viable solution for disaster-preparedness. | Andreatta PB, Maslowski E, Petty S, Shim W, Marsh M, Hall T, Stern S, Frankel J | Acad Emerg Med. 2010 Aug;17(8):870-6. doi: 10.1111/j.1553-2712.2010.00728.x. | 3 |
| 2010 | Virtual patient simulation: knowledge gain or knowledge loss? | Botezatu M, Hult H, Tessma MK, Fors U | Med Teach. 2010;32(7):562-8. doi: 10.3109/01421590903514630. | 2 |
| 2010 | Restoration of Guyton´s diagram for regulation of the circulation as a basis for quantitative physiological model development. | Kofránek J, Rusz J | Physiol Res. 2010;59(6):897-908. Epub 2010 Jun 9. | 4 |
| 2010 | Virtual patient design and curricular integration evaluation toolkit. | Huwendiek S, de Leng BA | Med Educ. 2010 May;44(5):519. doi: 10.1111/j.1365-2923.2010.03665.x. No abstract available. | other |
| 2010 | Virtual patients in geriatric education. | Tan ZS, Mulhausen PL, Smith SR, Ruiz JG | Gerontol Geriatr Educ. 2010;31(2):163-73. doi: 10.1080/02701961003795813. | other |
| 2010 | Blended learning using virtual patients and skills laboratory training. | Lehmann R, Bosse HM, Huwendiek S | Med Educ. 2010 May;44(5):521-2. doi: 10.1111/j.1365-2923.2010.03653.x. Epub 2010 Mar 30. No abstract available. | T1&2E4 |
| 2010 | Virtual patients: ED-2 band-aid or valuable asset in the learning portfolio? | Tworek J, Coderre S, Wright B, McLaughlin K | Acad Med. 2010 Jan;85(1):155-8. doi: 10.1097/ACM.0b013e3181c4f8bf. | other |
| 2010 | Virtual patients as a practical realisation of the e-learning idea in medicine | Kononowicz AA, Hege I | book chapter in Soomro, Safeeullah, "E-Learning Experiences and Future" | other |
| 2010 | Promoting occupational health in secondary schools through virtual patients. [References]. | Wengenroth L, Hege I, Forderreuther K, Riu E, Mandl H, Kujath P, Radon K | Computers & Education. Vol.55(4), Dec 2010, pp. 1443-1448. | 1 |
| 2010 | Preparing for practice: Issues in virtual medical education. [References]. | Ellaway RH, Topps D | Wankel, Charles [Ed]; Malleck, Shaun [Ed]. (2010). Emerging ethical issues of life in virtual worlds. (pp. 101-117). viii, 222 pp. Charlotte, NC, US: Information Age Publishing; US. | other |
| 2010 | Virtual agents based simulation for training healthcare workers in hand hygiene procedures | Bertrand J, Babu SV, Polgreen P, Segre A | Lecture Notes in Computer Science (including subseries Lecture Notes in Artificial Intelligence and Lecture Notes in Bioinformatics) 6356 LNAI():125-131; 2010 | T3E4 |
| 2010 | Collaborative virtual environment model for medical E-learning | El-Razek SMA, El-Bakry HM, El-Wahed WFA, Mastorakis N | Proceedings of the 9th WSEAS International Conference on Applied Computer and Applied Computational Science, ACACOS '10 ():191-195 | other |
| 2010 | High score! - Motivation strategies for user participation in virtual human development | Halan S, Rossen B, Cendan J, Lok B | Lecture Notes in Computer Science (including subseries Lecture Notes in Artificial Intelligence and Lecture Notes in Bioinformatics) 6356 LNAI():482-488 | 7 |
| 2010 | Virtual patients and online video resources: Useful when patient availability is reduced? | Hardy S, Brown G | The Clinical Teacher 7(4):292-293 | other |
| 2010 | Medical education on an interactive surface | Kaschny M, Buron S, Von Zadow U, Sostmann K | ACM International Conference on Interactive Tabletops and Surfaces, ITS 2010 ():267-268 | 4 |
| 2010 | Aspects of metacognitive self-awareness in Maryland virtual patient | Nirenburg S, McShane M, Beale S | AAAI Fall Symposium - Technical Report FS-10-01():69-74 | T4E2 |
| 2010 | Four kinds of learning in one agent-oriented environment | Nirenburg S, McShane M, Beale S, English J, Catizone R | Frontiers in Artificial Intelligence and Applications 221():92-97 | T4E2 |
| 2010 | Using virtual humans to bootstrap the creation of other virtual humans | Rossen B, Cendan J, Lok B | Lecture Notes in Computer Science (including subseries Lecture Notes in Artificial Intelligence and Lecture Notes in Bioinformatics) 6356 LNAI():392-398 | 7 |
| 2010 | Case-based medical E-assessment system | Scarlat R, Stanescu L, Popescu E, Burdescu DD | Proceedings - 10th IEEE International Conference on Advanced Learning Technologies, ICALT 2010 ():158-162 | 2 |
| 2010 | Real-time and realistic simulation for cardiac intervention with GPU | Yu R, Zhang S, Chiang P, Cai Y, Zheng J | ICCMS 2010 - 2010 International Conference on Computer Modeling and Simulation 3():68-72 | 4 |
| 2011 | Family physicians' completion of scoring criteria in Virtual Patient encounters. | Sumner W 2nd, O'Neill TR, Roussel G, Xu JZ, Fu H, Ivins D, Hagen MD | AMIA Annu Symp Proc. 2011;2011:1355-60. Epub 2011 Oct 22. | 2 |
| 2011 | A cognitive architecture for simulating bodies and minds. | Nirenburg S, McShane M, Beale S, Catizone R | AMIA Annu Symp Proc. 2011;2011:905-14. Epub 2011 Oct 22. | T4E2 |
| 2011 | Virtual patients: an effective educational intervention to improve paediatric basic specialist trainee education in the management of suspected child abuse? | McEvoy MM, Butler B, MacCarrick G, Nicholson AJ | Ir Med J. 2011 Sep;104(8):250-2. | 2 |
| 2011 | Virtual patients: a year of change. | Poulton T, Balasubramaniam C | Med Teach. 2011;33(11):933-7. doi: 10.3109/0142159X.2011.613501. | other |
| 2011 | Assessment of competencies by use of virtual patient technology. | Williams K, Wryobeck J, Edinger W, McGrady A, Fors U, Zary N | Acad Psychiatry. 2011 Fall;35(5):328-30. doi: 10.1176/appi.ap.35.5.328. No abstract available. | 2 |
| 2011 | Student perception of two different simulation techniques in oral and maxillofacial surgery undergraduate training. | Lund B, Fors U, Sejersen R, Sallnäs EL, Rosén A | BMC Med Educ. 2011 Oct 12;11:82. doi: 10.1186/1472-6920-11-82. | 2 |
| 2011 | A virtual surgery in general practice: evaluation of a novel undergraduate virtual patient learning package. | Gormley GJ, McGlade K, Thomson C, McGill M, Sun J | Med Teach. 2011;33(10):e522-7. doi: 10.3109/0142159X.2011.599889. | 2 |
| 2011 | Implementation of a web-based interactive virtual patient case simulation as a training and assessment tool for medical students. | Oliven A, Nave R, Gilad D, Barch A | Stud Health Technol Inform. 2011;169:233-7. | 2 |
| 2011 | Push and pull models to manage patient consent and licensing of multimedia resources in digital repositories for case-based reasoning. | Kononowicz AA, Zary N, Davies D, Heid J, Woodham L, Hege I | Stud Health Technol Inform. 2011;169:203-7. | other |
| 2011 | Acceptance of medical training cases as supplement to lectures. | Hörnlein A, Mandel A, Ifland M, Lüneberg E, Deckert J, Puppe F | GMS Z Med Ausbild. 2011;28(3):Doc42. doi: 10.3205/zma000754. Epub 2011 Aug 8. | 2 |
| 2011 | Simulated interviews 3.0: virtual humans to train abilities of diagnosis--usability assessment. | Peñaloza-Salazar C, Gutierrez-Maldonado J, Ferrer-Garcia M, Garcia-Palacios A, Andres-Pueyo A, Aguilar-Alonso A | Stud Health Technol Inform. 2011;167:165-9. | 7 |
| 2011 | Construct validity and reliability of structured assessment of endoVascular expertise in a simulated setting. | Bech B, Lönn L, Falkenberg M, Bartholdy NJ, Räder SB, Schroeder TV, Ringsted C | Eur J Vasc Endovasc Surg. 2011 Oct;42(4):539-48. doi: 10.1016/j.ejvs.2011.05.003. Epub 2011 Jun 15. | 4 |
| 2011 | Experiencing virtual patients in clinical learning: a phenomenological study. | Edelbring S, Dastmalchi M, Hult H, Lundberg IE, Dahlgren LO | Adv Health Sci Educ Theory Pract. 2011 Aug;16(3):331-45. doi: 10.1007/s10459-010-9265-0. Epub 2011 Jun 9. | 2 |
| 2011 | A collaborative model for developing and maintaining virtual patients for medical education. | Berman NB, Fall LH, Chessman AW, Dell MR, Lang VJ, Leong SL, Nixon LJ, Smith S | Med Teach. 2011;33(4):319-24. doi: 10.3109/0142159X.2011.540268. | other |
| 2011 | Design for learning: deconstructing virtual patient activities. | Ellaway RH, Davies D | Med Teach. 2011;33(4):303-10. doi: 10.3109/0142159X.2011.550969. | other |
| 2011 | How we created virtual patient cases for primary care-based learning. | Adams EC, Rodgers CJ, Harrington R, Young MD, Sieber VK | Med Teach. 2011;33(4):273-8. doi: 10.3109/0142159X.2011.544796. No abstract available. | 2 |
| 2011 | Implementation of virtual patients in the training for occupational health in Latin America. | Radon K, Carvalho D, Calvo MJ, Struempell S, Herrera V, Wengenroth L, Kausel G, Marchetti N, Rojas DS, Russ P, Hege I | Int J Occup Environ Health. 2011 Jan-Mar;17(1):63-70. | 2 |
| 2011 | Single and multi-user virtual patient design in the virtual world. | Taylor D, Patel V, Cohen D, Aggarwal R, Kerr K, Sevdalis N, Batrick N, Darzi A | Stud Health Technol Inform. 2011;163:650-2. | 3 |
| 2011 | Patient-specific cases for an ultrasound training simulator. | Petrinec K, Savitsky E, Hein C | Stud Health Technol Inform. 2011;163:447-53. | 4 |
| 2011 | Implementation of virtual online patient simulation. | Patel V, Aggarwal R, Taylor D, Darzi A | Stud Health Technol Inform. 2011;163:440-6. | 3 |
| 2011 | CliniSpace: a multiperson 3D online immersive training environment accessible through a browser. | Dev P, Heinrichs WL, Youngblood P | Stud Health Technol Inform. 2011;163:173-9. | 3 |
| 2011 | Mixed virtual reality simulation--taking endoscopic simulation one step further. | Courteille O, Felländer-Tsai L, Hedman L, Kjellin A, Enochsson L, Lindgren G, Fors U | Stud Health Technol Inform. 2011;163:144-6. | 4 |
| 2011 | Virtual patients for assessment of clinical reasoning in nursing -- a pilot study. | Forsberg E, Georg C, Ziegert K, Fors U | Nurse Educ Today. 2011 Nov;31(8):757-62. doi: 10.1016/j.nedt.2010.11.015. Epub 2010 Dec 14. | 2 |
| 2011 | Impact of genetic causal information on medical students' clinical encounters with an obese virtual patient: health promotion and social stigma. | Persky S, Eccleston CP | Ann Behav Med. 2011 Jun;41(3):363-72. doi: 10.1007/s12160-010-9242-0. | T3E7 |
| 2011 | Using a virtual patient activity to teach nurse prescribing. | Hurst HM, Marks-Maran D | Nurse Educ Pract. 2011 May;11(3):192-8. doi: 10.1016/j.nepr.2010.08.008. Epub 2010 Sep 24. | 2 |
| 2011 | Medical student bias and care recommendations for an obese versus non-obese virtual patient. | Persky S, Eccleston CP. | Int J Obes (Lond). 2011 May;35(5):728-35. doi: 10.1038/ijo.2010.173. Epub 2010 Sep 7. | T3E7 |
| 2011 | The influence of VERTâ„¢ characteristics on the development of skills in skin apposition techniques. | Green D, Appleyard R | Radiography 17(3): 178-82, 2011 | 4 |
| 2011 | eMedical teacher: Is it ok to say 'no'? [References]. | Ellaway R | Medical Teacher. Vol.33(1), Jan 2011, pp. 88-90. | other |
| 2011 | Technology innovations for behavioral education. | Gregerson MB | (2011). Technology innovations for behavioral education. xvii, 72 pp. New York, NY, US: Springer Science + Business Media; US. | other |
| 2011 | Learning retention of thoracic pedicle screw placement using a high-resolution augmented reality simulator with haptic feedback | Luciano CJ, Banerjee PP, Bellotte B, Oh GM, Lemole Jr M, Charbel FT, Roitberg B | Neurosurgery (2011) 69:SUPPL. 1 (ons14-ons19). Date of Publication: September 2011 | 4 |
| 2011 | Description of web-enhanced virtual character simulation system to standardize patient hand-offs | Filichia L, Halan S, Blackwelder E, Rossen B. Lok B, Korndorffer J, Cendan J | Journal of Surgical Research (2011) 166:2 (176-181). Date of Publication: April 2011 | 7 |
| 2011 | Development of an interactive application for learning medical procedures and clinical decision making | Bloice M, Simonic KM, Kreuzthaler M, Holzinger A | Lecture Notes in Computer Science (including subseries Lecture Notes in Artificial Intelligence and Lecture Notes in Bioinformatics) 7058 LNCS():211-224 | 2 |
| 2011 | Integrating haptics with augmented reality in a femoral palpation and needle insertion training simulation | Coles TR, John NW, Gould D, Caldwell DG | IEEE Transactions on Haptics 4(3):199-209 | 4 |
| 2011 | PATSy & VL-PATSy: Online case-based training for healthcare professionals | Cox R | AAAI Spring Symposium - Technical Report SS-11-01():41-44 | 2 |
| 2011 | Facilitating interoperability and standards for simulators in healthcare | Goldiez B, Zawadski R, Anton J, Anton EB, Garrity P, Sotomayor T | Spring Simulation Interoperability Workshop 2011, 2011 Spring SIW ():129-148 | other |
| 2011 | A review of information technology use in medical education: An overview | Luanrattana R | Proceedings - 7th International Conference on Information Processing and Management, ICIPM 2011 ():121-124 | other |
| 2011 | Dr. Vicky: A virtual coach for learning brief negotiated interview techniques for treating emergency room patients | Magerko B., Dean J, Idnani A, Pantalon M, D'Onofrio G | AAAI Spring Symposium - Technical Report SS-11-01():25-32 | 7 |
| 2011 | Can culture translate to the virtual world? | Sakpal R, Wilson DM | Communications in Computer and Information Science 173 CCIS(PART 1):242-246 | 7 |
| 2011 | Design and development of a Component-Based System for virtual patients in the virtual world of Second Life® | Toro-Troconis M, Kamat A, Partridge MR | Journal of Emerging Technologies in Web Intelligence 3(4):308-316 | 3 |
| 2011 | Interactive biochemistry - Sofia (IBS) - A flexible Web-based e-learning system | Trichkova E, Kossekova G | Proceedings of the IADIS International Conference e-Learning 2011, Part of the IADIS Multi Conference on Computer Science and Information Systems 2011, MCCSIS 2011 1():3-10 | 2 |
| 2012 | Preferred question types for computer-based assessment of clinical reasoning: a literature study. | van Bruggen L, Manrique-van Woudenbergh M, Spierenburg E, Vos J | Perspect Med Educ. 2012 Nov;1(4):162-71. doi: 10.1007/s40037-012-0024-1. Epub 2012 Oct 2. | other |
| 2012 | Development and preliminary evaluation of student-authored electronic cases. | Trace C, Baillie S, Short N | J Vet Med Educ. 2012 Winter;39(4):368-74. doi: 10.3138/jvme.0212-017R. | 2 |
| 2012 | Effectiveness of a virtual patient program in a psychiatry clerkship. | Lin CC, Wu WC, Liaw HT, Liu CC | Med Educ. 2012 Nov;46(11):1111-2. doi: 10.1111/medu.12020. No abstract available. | 2 |
| 2012 | Autonomous virtual patients in dentistry: system accuracy and expert versus novice comparison. | Clark GT, Suri A, Enciso R | J Dent Educ. 2012 Oct;76(10):1365-70. | T3E7 |
| 2012 | Use of virtual patients in dental education: a survey of U.S. and Canadian dental schools. | Cederberg RA, Bentley DA, Halpin R, Valenza JA | J Dent Educ. 2012 Oct;76(10):1358-64. | other |
| 2012 | An online virtual-patient program to teach pharmacists and pharmacy students how to provide diabetes-specific medication therapy management. | Battaglia JN, Kieser MA, Bruskiewitz RH, Pitterle ME, Thorpe JM | Am J Pharm Educ. 2012 Sep 10;76(7):131. doi: 10.5688/ajpe767131. | 2 |
| 2012 | Virtual patients as novel teaching tools in psychiatry. | Pataki C, Pato MT, Sugar J, Rizzo AS, Parsons TD, St George C, Kenny P | Acad Psychiatry. 2012 Sep 1;36(5):398-400. doi: 10.1176/appi.ap.10080118. No abstract available. | 7 |
| 2012 | An educational training simulator for advanced perfusion techniques using a high-fidelity virtual patient model. | Tokaji M, Ninomiya S, Kurosaki T, Orihashi K, Sueda T | Artif Organs. 2012 Dec;36(12):1026-35. doi: 10.1111/j.1525-1594.2012.01512.x. Epub 2012 Sep 11. | 4 |
| 2012 | NYU3T: teaching, technology, teamwork: a model for interprofessional education scalability and sustainability. | Djukic M, Fulmer T, Adams JG, Lee S, Triola MM | Nurs Clin North Am. 2012 Sep;47(3):333-46. doi: 10.1016/j.cnur.2012.05.003. Epub 2012 Jul 11. | 2 |
| 2012 | European pharmacy students' experience with virtual patient technology. | Cavaco AM, Madeira F | Am J Pharm Educ. 2012 Aug 10;76(6):106. doi: 10.5688/ajpe766106. | other |
| 2012 | Virtual patient simulation: a comparison of two approaches for capacity building in Sub-Saharan Africa. | Bediang G, Raetzo MA, Geissbuhler A | Stud Health Technol Inform. 2012;180:978-82. | 2 |
| 2012 | New approaches to linking clinical guidelines to virtual patients. | Kononowicz AA, Hege I, Krawczyk P, Zary N | Stud Health Technol Inform. 2012;180:958-62. | 2 |
| 2012 | Criteria to assess the quality of virtual patients. | Hege I, Zary N, Kononowicz AA | Stud Health Technol Inform. 2012;180:954-7. | other |
| 2012 | Virtual patients design and its effect on clinical reasoning and student experience: a protocol for a randomised factorial multi-centre study. | Bateman J, Allen ME, Kidd J, Parsons N, Davies D | BMC Med Educ. 2012 Aug 1;12:62. doi: 10.1186/1472-6920-12-62. | 2 |
| 2012 | The virtual continuity in learning programme: results. | Wood E, Tso S | Clin Teach. 2012 Aug;9(4):216-21. doi: 10.1111/j.1743-498X.2012.00551.x. | 2 |
| 2012 | Virtual patients in pharmacy education. | Jabbur-Lopes MO, Mesquita AR, Silva LM, De Almeida Neto A, Lyra DP Jr | Am J Pharm Educ. 2012 Jun 18;76(5):92. doi: 10.5688/ajpe76592. Review. | other |
| 2012 | Effects of introducing a voluntary virtual patient module to a basic life support with an automated external defibrillator course: a randomised trial. | Kononowicz AA, Krawczyk P, Cebula G, Dembkowska M, Drab E, Frączek B, Stachoń AJ, Andres J | BMC Med Educ. 2012 Jun 18;12:41. doi: 10.1186/1472-6920-12-41. | 2 |
| 2012 | Novel educational approach for medical students: improved retention rates using interactive medical software compared with traditional lecture-based format. | Subramanian A, Timberlake M, Mittakanti H, Lara M, Brandt ML | J Surg Educ. 2012 Jul;69(4):449-52. doi: 10.1016/j.jsurg.2012.05.013. | 2 |
| 2012 | evPaeds: undergraduate clinical reasoning. | Pinnock R, Spence F, Chung A, Booth R | Clin Teach. 2012 Jun;9(3):152-7. doi: 10.1111/j.1743-498X.2011.00523.x. | 2 |
| 2012 | Interactive virtual-patient scenarios: an evolving tool in psychiatric education. | Shah H, Rossen B, Lok B, Londino D, Lind SD, Foster A | Acad Psychiatry. 2012 Mar 1;36(2):146-50. doi: 10.1176/appi.ap.10030049. No abstract available. | 7 |
| 2012 | Virtual patients for assessment of medical student ability to integrate clinical and laboratory data to develop differential diagnoses: comparison of results of exams with/without time constraints. | Gunning WT, Fors UG | Med Teach. 2012;34(4):e222-8. doi: 10.3109/0142159X.2012.642830. | 2 |
| 2012 | Integrating virtual patients into courses: follow-up seminars and perceived benefit. | Edelbring S, Broström O, Henriksson P, Vassiliou D, Spaak J, Dahlgren LO, Fors U, Zary N | Med Educ. 2012 Apr;46(4):417-25. doi: 10.1111/j.1365-2923.2012.04219.x. | 2 |
| 2012 | Virtual patients can be used to teach clinical reasoning. | Bateman J, Hariman C, Nassrally M | Clin Teach. 2012 Apr;9(2):133-4. doi: 10.1111/j.1743-498X.2012.00546.x. No abstract available. | other |
| 2012 | The use of virtual patients in medical school curricula. | Cendan J, Lok B | Adv Physiol Educ. 2012 Mar;36(1):48-53. doi: 10.1152/advan.00054.2011. Review. | other |
| 2012 | Shader Lamps Virtual Patients: the physical manifestation of virtual patients. | Rivera-Gutierrez D, Welch G, Lincoln P, Whitton M, Cendan J, Chesnutt DA, Fuchs H, Lok B | Stud Health Technol Inform. 2012;173:372-8. | 4 |
| 2012 | Effectively training pediatric residents to deliver diagnoses of Down syndrome. | Lunney CA, Kleinert HL, Ferguson JE 2nd, Campbell L | Am J Med Genet A. 2012 Feb;158A(2):384-90. doi: 10.1002/ajmg.a.34422. Epub 2012 Jan 13. | 2 |
| 2012 | Can a virtual patient trainer teach student nurses how to save lives--teaching nursing students about pediatric respiratory diseases. | LeFlore JL, Anderson M, Zielke MA, Nelson KA, Thomas PE, Hardee G, John LD | Simul Healthc. 2012 Feb;7(1):10-7. doi: 10.1097/SIH.0b013e31823652de. | 3 |
| 2012 | Teaching professionalism through virtual means. | McEvoy M, Butler B, MacCarrick G | Clin Teach. 2012 Feb;9(1):32-6. doi: 10.1111/j.1743-498X.2011.00487.x. | 5 |
| 2012 | Predictors of primary care physicians' self-reported intention to conduct suicide risk assessments. | Hooper LM, Epstein SA, Weinfurt KP, DeCoster J, Qu L, Hannah NJ | J Behav Health Serv Res. 2012 Apr;39(2):103-15. doi: 10.1007/s11414-011-9268-5. | 5 |
| 2012 | What can virtual patient simulation offer mental health nursing education? | Guise V, Chambers M, Välimäki M | J Psychiatr Ment Health Nurs. 2012 Jun;19(5):410-8. doi: 10.1111/j.1365-2850.2011.01797.x. Epub 2011 Nov 1. | 2 |
| 2012 | Development, implementation and initial evaluation of narrative virtual patients for use in vocational mental health nurse training. | Guise V, Chambers M, Conradi E, Kavia S, Välimäki M | Nurse Educ Today. 2012 Aug;32(6):683-9. doi: 10.1016/j.nedt.2011.09.004. Epub 2011 Nov 4. | 2 |
| 2012 | Efficacy of Virtual Patients in Medical Education: A Meta-Analysis of Randomized Studies | Consorti F, Mancuso R, Nocioni M, Piccolo A | Computers & Education,59,3,1001--1008,2012 | other |
| 2012 | Making a case: Validating criterion-referenced guidelines for virtual patient case authoring. | Posel N | Dissertation Abstracts International Section A: Humanities and Social Sciences. Vol.73(2-A),2012, pp. 490. | other |
| 2012 | Creating contextual learning experiences via virtual simulation. | Yehle RF | Dissertation Abstracts International Section A: Humanities and Social Sciences. Vol.72(9-A),2012, pp. 3151. | 3 |
| 2012 | Evaluating the effectiveness of Virtual Patients to promote clinical reasoning. | Wilson JI | Dissertation Abstracts International Section A: Humanities and Social Sciences. Vol.72(9-A),2012, pp. 3235. | 2 |
| 2012 | A crowdsourcing method to develop virtual human conversational agents. [References]. | Rossen B, Lok B | International Journal of Human-Computer Studies. Vol.70(4), Apr 2012, pp. 301-319. | 7 |
| 2012 | Case creation and e-learning in a web-based virtual department of urology using the INMEDEA simulator | Horstmann M, Horstmann C, Renninger M | Nephro-Urology Monthly (2012) 4:1 (356-360). Date of Publication: 2012 | 2 |
| 2012 | Virtual patient simulation: Promotion of clinical reasoning abilities of medical students | Aghili O, Khamseh ME, Taghavinia M, Malek M, Emami Z, Baradaran HR, Mafinejad MK | Knowledge Management and E-Learning 2012 4(4):518-527 | 2 |
| 2012 | Assessing past, present, and future interactions with virtual patients | Ferdig RE, Schottke K, Rivera-Gutierrez D, Lok B | International Journal of Gaming and Computer-Mediated Simulations 4(3):20-37 | other |
| 2012 | Virtual human personality masks: A human computation approach to modeling verbal personalities in virtual humans | Krishnan V, Foster A, Kopper R, Lok B | Lecture Notes in Computer Science (including subseries Lecture Notes in Artificial Intelligence and Lecture Notes in Bioinformatics) 7502 LNAI():146-152 | 7 |
| 2012 | Design and validation of an epidural needle insertion simulator with haptic feedback for training resident anaesthesiologists | Manoharan V, Van Gerwen D, Van Den Dobbelsteen JJ, Dankelman J | Haptics Symposium 2012, HAPTICS 2012 - Proceedings ():341-348 | 4 |
| 2012 | MV-SYDIME: A virtual patient for medical diagnosis apprenticeship | Monthe V, Batchakui B, Tangha C | International Journal of Online Engineering 8(4):21-31 | T4E2 |
| 2012 | Virtual patients to teach cultural competency | Sakpal R | ICMI'12 - Proceedings of the ACM International Conference on Multimodal Interaction ():349-352 | 7 |
| 2012 | Introduction of clinical, simulation-based software for medical sciences teachings | Shahbazi B, Edalat-Nejad M, Edalat-Nejad N, Edalatnejad M | Procedia Engineering 29():43-47 | 2 |
| 2012 | Sorting out the virtual patient: How to exploit artificial intelligence, game technology and sound educational practices to create engaging role-playing simulations | Talbot TB, Sagae K, John B, Rizzo AA | International Journal of Gaming and Computer-Mediated Simulations 4(3):1-19 | other |
| 2013 | Virtual patient design: exploring what works and why. A grounded theory study. | Bateman J, Allen M, Samani D, Kidd J, Davies D | Med Educ. 2013 Jun;47(6):595-606. doi: 10.1111/medu.12151. | 2 |
| 2013 | The virtual-patient pilot: testing a new tool for undergraduate surgical education and assessment. | Yang RL, Hashimoto DA, Predina JD, Bowens NM, Sonnenberg EM, Cleveland EC, Lawson C, Morris JB, Kelz RR | J Surg Educ. 2013 May-Jun;70(3):394-401. doi: 10.1016/j.jsurg.2012.12.001. | 2 |
| 2013 | What are effects of a spaced activation of virtual patients in a pediatric course? | Maier EM, Hege I, Muntau AC, Huber J, Fischer MR | BMC Med Educ. 2013 Mar 28;13:45. doi: 10.1186/1472-6920-13-45. | 2 |
| 2013 | Systematic Review of Effectiveness of Situated E-Learning on Medical and Nursing Education. | Feng JY, Chang YT, Chang HY, Erdley WS, Lin CH, Chang YJ | Worldviews Evid Based Nurs. 2013 Mar 19. doi: 10.1111/wvn.12005. [Epub ahead of print] | other |
| 2013 | Adaptive space warping to enhance passive haptics in an arthroscopy surgical simulator. | Spillmann J, Tuchschmid S, Harders M | IEEE Trans Vis Comput Graph. 2013 Apr;19(4):626-33. doi: 10.1109/TVCG.2013.23. | 4 |
| 2013 | An innovative blended learning approach using virtual patients as preparation for skills laboratory training: perceptions of students and tutors. | Lehmann R, Bosse HM, Simon A, Nikendei C, Huwendiek S | BMC Med Educ. 2013 Feb 12;13:23. doi: 10.1186/1472-6920-13-23. | T1&2E4 |
| 2013 | Face validity of VIS-Ed: a visualization program for teaching medical students and residents the biomechanics of cervical spine trauma. | Courteille O, Ho J, Fahlstedt M, Fors U, Felländer-Tsai L, Hedman L, Möller H | Stud Health Technol Inform. 2013;184:96-102. | 4 |
| 2013 | Language, culture and international exchange of virtual patients. | Muntean V, Calinici T, Tigan S, Fors UG | BMC Med Educ. 2013 Feb 11;13:21. doi: 10.1186/1472-6920-13-21. | 2 |
| 2013 | Clinical decision making: a pilot e-learning study. | Abendroth M, Harendza S, Riemer M | Clin Teach. 2013 Feb;10(1):51-5. doi: 10.1111/j.1743-498X.2012.00629.x. | 2 |
| 2013 | Optimal learning in a virtual patient simulation of cranial nerve palsies: the interaction between social learning context and student aptitude. | Johnson TR, Lyons R, Chuah JH, Kopper R, Lok BC, Cendan JC | Med Teach. 2013;35(1):e876-84. doi: 10.3109/0142159X.2012.714884. Epub 2012 Sep 3. | 7 |
| 2013 | Blended learning in surgery using the Inmedea Simulator. | Funke K, Bonrath E, Mardin WA, Becker JC, Haier J, Senninger N, Vowinkel T, Hoelzen JP, Mees ST | Langenbecks Arch Surg. 2013 Feb;398(2):335-40. doi: 10.1007/s00423-012-0987-8. Epub 2012 Aug 10. | 2 |
| 2013 | New directions in e-learning research in health professions education: Report of two symposia. | Triola MM, Huwendiek S, Levinson AJ, Cook DA | Med Teach. 2012;34(1):e15-20. doi: 10.3109/0142159X.2012.638010. | other |
| 2013 | Percutaneous spinal fixation simulation with virtual reality and haptics | Luciano CJ, Banerjee PP, Sorenson JM, Foley KT, Ansari SA, Rizzi S, Germanwala AV, Kranzler L, Chittiboina P, Roitberg BZ | Neurosurgery (2013) 72:SUPPL. 1 (A89-A96). Date of Publication: January 2013 | 4 |
| 2013 | Skills of primary healthcare physicians in paediatric cardiac auscultation | Germanakis I, Petridou ET, Varlamis G, Matsoukis IL, Papadopoulou-Legbelou K, Kalmanti M | Acta Paediatrica, International Journal of Paediatrics (2013) 102:2 (e74-e78). Date of Publication: February 2013 | T1&2E4 |
| 2013 | SimMed: Combining simulation and interactive tabletops for medical education | Von Zadow U., Buron S., Harms T., Behringer F., Sostmann K., Dachselt R | Conference on Human Factors in Computing Systems - Proceedings ():1469-1478 | 4 |
| 2013 | A virtual patient software program to improve pharmacy student learning in a comprehensive disease management course. | Douglass MA, Casale JP, Skirvin JA, Divall MV | Am J Pharm Educ. 2013 Oct 14;77(8):172. doi: 10.5688/ajpe778172. | 2 |
| 2013 | Cost considerations in using simulations for medical training. | Fletcher JD, Wind AP | Mil Med. 2013 Oct;178(10 Suppl):37-46. doi: 10.7205/MILMED-D-13-00258. | other |
| 2013 | The Evolving Role of Online Virtual Patients in Internal Medicine Clerkship Education Nationally. | Lang VJ, Kogan J, Berman N, Torre D | Acad Med. 2013 Sep 25. [Epub ahead of print] | 2 |
| 2013 | Promotion of Self-directed Learning Using Virtual Patient Cases. | Benedict N, Schonder K, McGee J | Am J Pharm Educ. 2013 Sep 12;77(7):151. doi: 10.5688/ajpe777151. | 2 |
| 2013 | On the usage of health records for the design of virtual patients: a systematic review. | Bloice MD, Simonic KM, Holzinger A | BMC Med Inform Decis Mak. 2013 Sep 8;13(1):103. [Epub ahead of print] | other |
| 2013 | Learner preferences regarding integrating, sequencing and aligning virtual patients with other activities in the undergraduate medical curriculum: A focus group study. | Huwendiek S, Duncker C, Reichert F, De Leng BA, Dolmans D, van der Vleuten CP, Haag M, Hoffmann GF, Tönshoff B | Med Teach. 2013 Nov;35(11):920-9. doi: 10.3109/0142159X.2013.826790. Epub 2013 Sep 4. | 2 |
| 2013 | Integration of problem-based learning and innovative technology into a self-care course. | McFalls M | Am J Pharm Educ. 2013 Aug 12;77(6):127. doi: 10.5688/ajpe776127. | 7 |
| 2013 | Educational potential of a virtual patient system for caring for traumatized patients in primary care. | Ekblad S, Mollica RF, Fors U, Pantziaras I, Lavelle J | BMC Med Educ. 2013 Aug 19;13:110. doi: 10.1186/1472-6920-13-110. | 7 |
| 2013 | Clinical reasoning in nursing, a think-aloud study using virtual patients - A base for an innovative assessment. | Forsberg E, Ziegert K, Hult H, Fors U | Nurse Educ Today. 2013 Jul 25. doi:pii: S0260-6917(13)00261-X. 10.1016/j.nedt.2013.07.010. [Epub ahead of print] | 2 |
| 2013 | Developing clinical skills using a virtual patient simulator in a resource-limited setting. | Bediang G, Franck C, Raetzo MA, Doell J, Ba M, Kamga Y, Baroz F, Geissbuhler A | Stud Health Technol Inform. 2013;192:102-6. | 2 |
| 2013 | A randomized trial of two e-learning strategies for teaching substance abuse management skills to physicians. | Harris JM Jr, Sun H | Acad Med. 2013 Sep;88(9):1357-62. doi: 10.1097/ACM.0b013e31829e7ec6. | 2 |
| 2013 | An integrative OSCE methodology for enhancing the traditional OSCE program at Taipei medical university ospital - a feasibility study. | Lin CW, Clinciu DL, Swartz MH, Wu CC, Lien GS, Chan CY, Lee FP, Li YC | BMC Med Educ. 2013 Jul 26;13(1):102. [Epub ahead of print] | 2 |
| 2013 | Implementation of an interactive virtual-world simulation for structured surgeon assessment of clinical scenarios. | Patel V, Aggarwal R, Cohen D, Taylor D, Darzi A | J Am Coll Surg. 2013 Aug;217(2):270-9. doi: 10.1016/j.jamcollsurg.2013.03.023. | 3 |
| 2013 | Interactive algorithms for teaching and learning acute medicine in the network of medical faculties MEFANET. | Schwarz D, Štourač P, Komenda M, Harazim H, Kosinová M, Gregor J, Hůlek R, Smékalová O, Křikava I, Štoudek R, Dušek L | J Med Internet Res. 2013 Jul 8;15(7):e135. doi: 10.2196/jmir.2590. | 2 |
| 2013 | Systematic Review of Effectiveness of Situated E-Learning on Medical and Nursing Education. | Feng JY, Chang YT, Chang HY, Erdley W, Scott L, Chyi-Her, Chang YJ | Worldviews on Evidence-Based Nursing 10(3): 174-183 | other |
| 2013 | Meet Mohammed: Using simulation and technology to support learning | Lambert N, Watkins L | Journal of Mental Health Training, Education and Practice: 8(2): 66-75 | 3 |
| 2013 | Death is not always a failure: outcomes from implementing an online virtual patient clinical case in palliative care for family medicine clerkship. | Tan A, Ross SP, Duerksen K | Med Educ Online. 2013 Nov 22;18:22711. doi: 10.3402/meo.v18i0.22711. | 2 |
| 2013 | Are students ready for meaningful use? | Ferenchick GS, Solomon D, Mohmand A, Towfiq B, Kavanaugh K, Warbasse L, Addison J, Chames F | Med Educ Online. 2013 Nov 19;18:22495. doi: 10.3402/meo.v18i0.22495. | 2 |
| 2013 | Developing virtual patients for medical microbiology education. | McCarthy D, O'Gorman C, Gormley GJ | Trends Microbiol. 2013 Dec;21(12):613-5. doi: 10.1016/j.tim.2013.10.002. Epub 2013 Nov 8. | 1 |
| 2013 | Design and evaluation of a simulation for pediatric dentistry in virtual worlds. | Papadopoulos L, Pentzou AE, Louloudiadis K, Tsiatsos TK | J Med Internet Res. 2013 Oct 29;15(11):e240. doi: 10.2196/jmir.2651. Erratum in: J Med Internet Res. 2013;15(11):e268. | T3E7 |
| 2013 | Medical Training in Transition: Interprofessional and Intercultural Training Enhanced by E-Learning Tools for Primary Healthcare Education: A Review. | Ekblad S, Fors UG, Salminen AA | International Medical Journal 20(6):644-645 | other |
| 2013 | Enhancing students' communication in an ethnic language. [References]. | Azam S, Carroll M | Medical Education. Vol.47(11), Nov 2013, pp. 1144-1145. | 5 |
| 2013 | Using critical-cue inventories to advance virtual patient technologies in psychological assessment | Morrison BW, Morrison NMV, Morton, J, Harris J | Proceedings of the 25th Australian Computer-Human Interaction Conference: Augmentation, Application, Innovation, Collaboration, OzCHI 2013: 531-534 | 7 |
| 2013 | Empirical evaluation of traditional vs. hybrid interaction metaphors in a multitask healthcare simulation | Dukes LC, Bertrand J, Gupta M, Armstrong R, Fasolino T, Babu S, Hodges, LF | Proceedings - 2013 IEEE International Conference on Healthcare Informatics, ICHI 2013: 89-98 | 3 |
| 2013 | An efficient virtual patient image model interview training in pharmacy | Park M, Summons P | International Journal of Bio-Science and Bio-Technology: 137-145 | 7 |
| 2013 | Are virtual patients effective to train diagnostic skills? A study with bulimia nervosa virtual patients | Gutierrez-Maldonado J, Ferrer-Garcia M | Proceedings of the ACM Symposium on Virtual Reality Software and Technology, VRST: 267 | T7E2 |
